# Lung cancer diagnosis through extracellular vesicle analysis using label-free surface-enhanced Raman spectroscopy coupled with machine learning

**DOI:** 10.7150/thno.110178

**Published:** 2025-06-23

**Authors:** Hai-Sha Liu, Kai-Wen Ye, Jun Liu, Jin-Kuang Jiang, Ying-Fang Jian, Dong-Mei Chen, Chao Kang, Li Qiu, Ya-Juan Liu

**Affiliations:** 1 School of Chemistry and Chemical Engineering, Guizhou University, Guiyang 550025, China.; 2 Department of Thoracic Surgery and Oncology, the First Affiliated Hospital of Guangzhou Medical University, State Key Laboratory of Respiratory Disease & National Clinical Research Center for Respiratory Disease, Guangzhou 510120, China.; 3 Guangzhou Municipal and Guangdong Provincial Key Laboratory of Molecular Target & Clinical Pharmacology, the NMPA and State Key Laboratory of Respiratory Disease, School of Pharmaceutical Sciences, Guangzhou Medical University, Guangzhou 511436, China.

**Keywords:** surface-enhanced Raman spectroscopy, extracellular vesicles, machine learning, deep learning, convolutional neural network

## Abstract

**Rationale:** Label-free surface-enhanced Raman spectroscopy (SERS) based on extracellular vesicles (EVs) has great potential in cancer diagnosis. However, the repeatability and stability of the SERS signals and the accurate early prediction of multiple cell types based on a small number of samples still require further research.

**Methods:** We developed a highly accurate classification approach to distinguish EVs derived from lung cancer and normal cells. This method was further validated using mixed samples of cell-derived EVs and plasma-derived EVs from both healthy and lung cancer mouse models and patients. The approach integrates label-free SERS analysis of EVs with machine learning techniques, including support vector machines (SVM) and convolutional neural networks (CNN), for robust classification. To preserve the native state of EVs, a capillary-based liquid-phase sampling method was employed, avoiding the need for drying. Additionally, the size and related properties of the SERS substrates were systematically optimized. Bayesian optimization was further applied to refine the SVM hyperparameters, enhancing classification performance.

**Results:** The classification error rate of the five-fold cross-validation (CVloss) of the SVM model (with hyperparameters optimized by Bayesian method) of A549 and BEAS-2B cell-derived EVs was 3.7%, and the overall accuracy of the independent test set reached 98.7%. The results of principal component analysis, the Shapley values and partial dependence plot analysis indicate higher levels of collagen and adenine in cancer cells compared to normal cells, this may be due to the large amount of collagen used as a source of nutrients in cancer cells and abnormal DNA or RNA metabolism. The overall accuracy of the test set predicted by the SVM and CNN models of plasma-derived EVs from lung cancer and healthy mice was 97.5 % and 95.8 %, respectively. Finally, the proposed strategy was used to discriminate plasma-derived EVs from lung cancer patients and healthy people, the CVloss of the SVM and CNN model was 7.7% and 8.3%, the overall accuracy of the independent test set was 91.5% and 95.4%, respectively.

**Conclusions:** The proposed machine learning-assisted, liquid-phase enhanced SERS method offers notable advantages, including minimal sample volume, high stability, and excellent accuracy. The promising classification performance demonstrates its potential as a rapid and reliable approach for the early detection and monitoring of lung cancer through clinical blood sample analysis.

## Introduction

Cancer is a major global health problem, and if detected at an early stage, timely medical intervention can be performed to slow down or prevent the spread and lesions of cancer. However, about 50% of cancers are found in the late stage 1-3. Therefore, the accurate identification of multiple cancers at an early stage is essential for diagnosis, timely intervention, and effective treatment. Circulating extracellular vesicles (EVs) in complex biological fluids contain proteins, mRNA, DNA fragments, noncoding RNA, and lipids, which are responsible for the transport of lipids, metabolites, nucleic acids, and nonmembrane and transmembrane proteins 4-6, and play a key role in intercellular communication 7, 8. Two mechanisms of their formation exist: one is formed by cells releasing lipid-bound vesicles into the extracellular matrix to interact with other cells 9, and the other is by sprouting directly from the plasma membrane 10. EVs can serve as valuable disease biomarkers for the diagnosis, prognosis, and monitoring of therapeutic responses in multiple disease states 11.

Detection of EVs is extremely difficult because they exist in complex biological samples 12. Classical methods for the detection of EVs include nanoparticle tracking analysis (NTA) 13, transmission electron microscopy (TEM) 14, western blot (WB) 15, and enzyme-linked immunosorbent assay (ELISA) 16. These methods require complex sample pretreatment, large sample volumes, and high costs, which are laborious and time-consuming, and greatly limit their use in EV identification and analytical applications. In recent years, many new methods for EV detection have been developed, including fluorescence 17, electrochemistry 18, and colorimetric methods 19, which are complementary to classical methods in some respects. Fluorescence methods have high sensitivity and selectivity, but they rely on efficient fluorophores and specific interaction sites on EVs. Electrochemical methods require moderate sample volumes and high accuracy, but their sensitivity is limited at low analyte concentrations. The colorimetric method is simple and has low requirements for equipment, but the error is large. Therefore, the establishment of a sensitive and accurate EV detection method is helpful in exerting the potential of EVs as essential biomarkers and in promoting their application in early screening of essential diseases such as cancer.

In response to the above scientific problems, researchers have explored various signal enhancement strategies to improve the sensitivity of EV detection. Among them, surface-enhanced Raman spectroscopy (SERS) can effectively amplify Raman signals, which has made a lot of progress in disease diagnosis and clinical bioimaging research 20-22. It is an ultrasensitive analytical method that is based on the principle of surface plasmon resonance to enhance the Raman signal of analytes using gelatinous metal nanoparticles such as silver and gold, or the rough surface of their two-dimensional metals 23, 24. In particular, the use of nanomaterials with high thermal conductivity, high adsorption capacity, high biocompatibility, and high specific surface area can improve the efficiency of electron transport and the loading capacity of signal molecules, thereby amplifying the specific fingerprint information of the detected substance and enhancing the Raman signal by several orders of magnitude, enabling trace analysis and even down to the single molecule level of analytes 24-29.

In recent years, more and more machine learning and deep learning algorithms have been combined with SERS, which has greatly improved spectral analysis methods and made a lot of progress in EV classification research. Principal component analysis (PCA) is often supplemented with more complex algorithms to obtain more accurate classification results. For example, Diao et al. successfully distinguished EVs from four types of cells using PCA-linear discriminant analysis combined with SERS spectroscopy, with an overall accuracy of 91.1% for cell-derived EVs 30. In addition, these algorithms include partial least squares discriminant analysis 31, support vector machines (SVM) 32, and K-nearest neighbor (KNN) 33. For example, Li et al. used PCA-SVM combined with SERS to predict HepG2, HeLa, 143B, LO-2, BMSC, and H8 cell-derived exosomes with an overall accuracy of 94.4% 34. Deep learning can handle large and complex spectral datasets and can provide more accurate results in various applications. For example, Shin et al. used a deep learning model to classify normal and lung cancer cell-derived EVs with an overall accuracy of 95.0% 35; The artificial intelligence model constructed by Shin et al. successfully identified six early-stage cancers with the area under curve (AUC) of 0.970 36; The ANN model constructed by Xie et al. 37 had a 100% classification accuracy for serum-derived exosome samples from breast cancer patients. Current advances in machine learning and deep learning algorithms have improved the accuracy of Raman spectral recognition. However, the accurate early prediction of various cancer cells based on a small number of EV samples is still worthy of further study.

Traditionally, EVs are air-dried before Raman analysis to eliminate residual liquid that could interfere with spectroscopic measurements, potentially causing structural changes or loss of function. If in solution, the heat generated by the laser can be dissipated more efficiently, preventing local heating, thus avoiding sample damage and thermal drift, which is conducive to improving the stability of SERS measurements. For possible spectral perturbations by liquids, correlation algorithms can be used to remove the background, and smoothing algorithms, such as Fourier transforms, can be used to improve the signal-to-noise ratio of the SERS 38-40.

**Figure 1:**
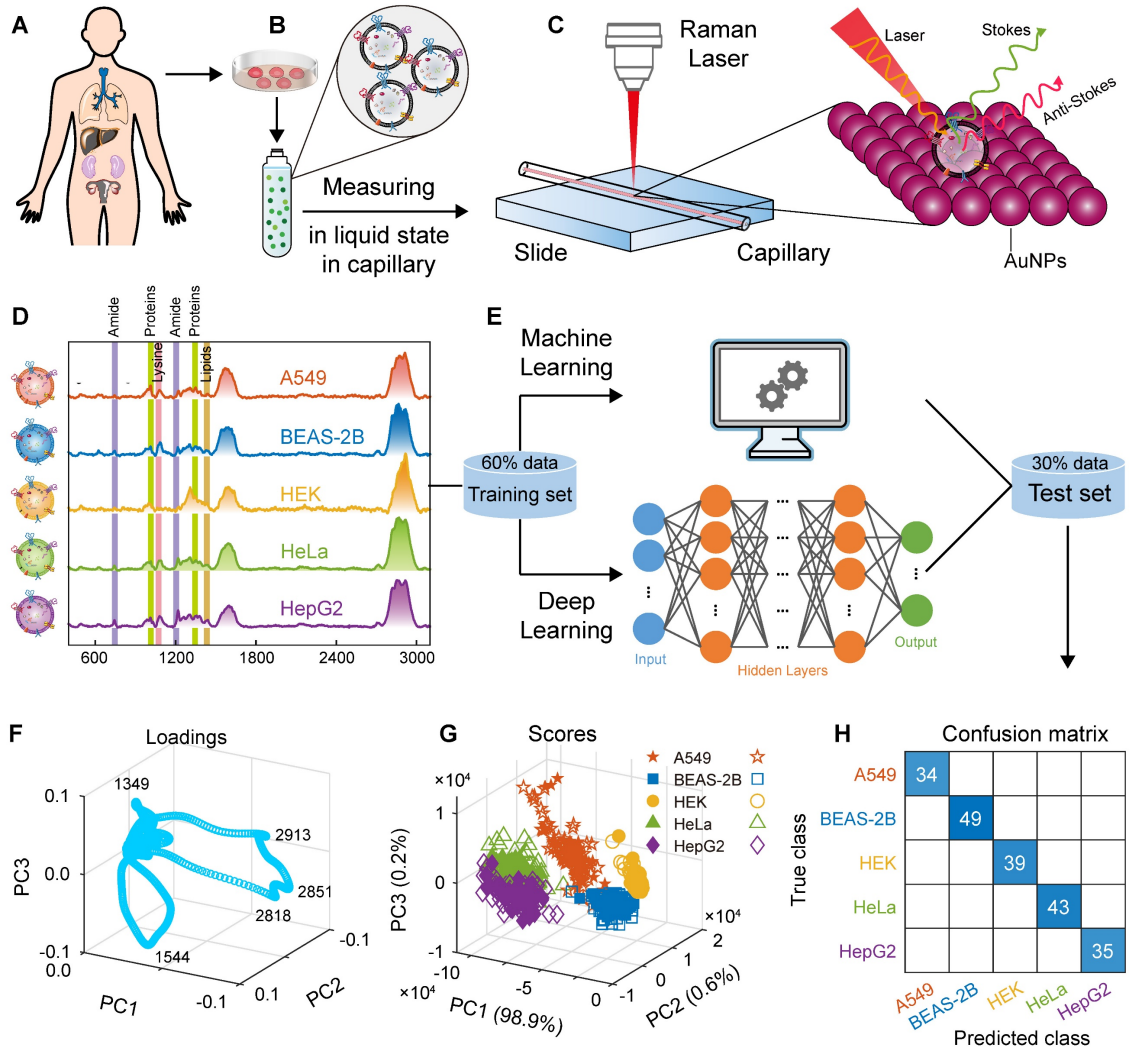
Schematic of the method flow. (A) Sampling. (B) Isolation of EVs. (C) Measurement of SERS. (D) Raman spectra. (E) Modelling. (F) Loadings of PCA. (G) Scores of PCA. (H) Confusion matrix.

In this study, we used a capillary-based liquid sampling method to produce exosomes in a liquid state without the need for drying. The size and related properties of the SERS substrate, including uniformity, reproducibility and enhancement effect were optimized. Thus, a stable SERS signal of exosomes was obtained. On the basis of these spectra combined with the PCA-SVM algorithm in machine learning (hyperparameters were optimized using a Bayesian method), a small sample accurate classification method was constructed for EVs from lung cancer cells and normal cells. To classify of multiple cell-derived EVs, we also designed and trained a convolutional neural network (CNN) architecture to construct a deep learning model, which successfully achieved high accuracy classification of five cell-derived EVs including human non-small cell lung cancer cells (A549), lung epithelial cells (BEAS-2B), embryonic kidney cells (HEK), cervical cancer cells (HeLa), and liver cancer cells (HepG2). The steps of this study are illustrated in Figure 1. We also obtained biochemical profiles in which higher levels of collagen and adenine were observed in cancer cells than in normal cells. In addition, the mixed samples of A549 and BEAS-2B cell-derived exosomes, animal samples, and real clinical samples were used to verify the predictive ability of the proposed method for EVs, respectively. This method is expected to provide an analytical strategy for liquid biopsy of lung cancer.

## Materials and Instruments

### Pharmaceuticals and reagents

Tetrachloroauric acid trihydrate (HAuCl_4_·3H_2_O) was purchased from Smart Biotechnology Co., Ltd. (Guangzhou, China). Sodium citrate dihydrate (C_6_H_5_Na_3_O_7_·2H_2_O), and Rhodamine 6G (C_28_H_31_N_2_O_3_Cl) were purchased from Aladdin Biochemical Technology Co., Ltd. (Shanghai, China). Hydrochloric acid (HCl), nitric acid (HNO_3_), sulfuric acid (H_2_SO_4_), and hydrogen peroxide (H_2_O_2_) were purchased from Chuandong Chemical (Group) Co., Ltd. (Chongqing, China). Uranium acetate was purchased from Taosheng Optoelectronics Technology Co., Ltd. (Shanghai, China). Phosphate buffer saline (PBS), SDS-PAGE Protein Loading Buffer (5X), and phosphate buffered saline with tween 20 (PBST) were purchased from Biyuntian Biotechnology Co., Ltd. (Shanghai, China). The chromogenic solution was purchased from Yaenzyme Biomedical Technology Co., Ltd. (Shanghai, China). The spotting capillary was purchased from Titan Technology Co., Ltd. (Shanghai, China) and is a transparent hard glass with a size of 0.5 mm × 100 mm. All reagents were analytically pure (AR) without further purification. Ultrapure water (with resistivity of 18.2 MΩ·cm) was used in the whole experiment.

### Cell culture

For the five types of cells, EVs were isolated by ultracentrifuge (OPTIMA XPN-80, Beckman, USA). After removing dead cells and cell debris from the cultured cells, the supernatant was filtered and concentrated, which was centrifuged at 100,000× g for 2 h, and the precipitate was collected. Then, the formed particles were resuspended in PBS and centrifuged at 100,000× g for 2 h. Finally, the obtained particles were resuspended in PBS and stored for later use.

### Blood sample from mice

For plasma collected from mice (the specific steps are shown in the ''Animal experiments of lung cancer and healthy mice'' section of the supporting material), the cells were removed by centrifugation at 4 °C, 300 g for 5 min, the cells and their fragments were further removed by centrifugation at 4 °C, 2000 g for 10 min, and the supernatant was retained. The particles were removed by centrifugation at 4 °C, 14000 g for 30 min, and the supernatant was collected. Exosomes were extracted using a magnetic bead exosome extraction kit. This part of the research was carried out on the sci-go instrument test platform (Beijing, China) and has been approved by the Experimental Animal Welfare and Ethics Committee of the Institute of General Health of Hefei Comprehensive National Science Center (No. IHM-AP-2025-006-R7).

### Clinical sample

For plasma samples collected from patients with early-stage (stage I-II) lung cancer and healthy participants (see the ''Clinical sample of lung cancer patients and healthy people'' section of the supporting material for specific steps), in the first step, serum was sampled and diluted 10 times with 0.9 % normal saline, and then the diluent was filtered with a 0.22 μm filter. The diluent was concentrated 10 times with a tangential flow device (300 kda) to collect the concentrate. In the second step, the concentrate was handled as the first step, the concentrate was collected and the protein concentration of the filtrate was detected. The second step was repeated until the protein concentration of the filtrate was 0, and the concentrated solution was serum exosomes. This part of the study has been approved by the Ethics Committee of Scientific Research Project Review of the First Affiliated Hospital of Guangzhou Medical University (No. ES-2025-K125-01), and all participants have signed informed consent.

### Characterization of EVs

The morphological sizes of EVs were observed using a biotransmission microscope (JEM 2100F, JEOL, Japan) at an accelerating voltage of 120 kV. The sample preparation method for this measurement was as follows: 5 μL of EVs was added dropwise to the copper mesh, the excess EVs were absorbed using filter paper after 1 min, then the EVs were negatively stained with 5 μL of 2% uranium acetate for 1 min, and the excess dye solution was removed using filter paper. After natural drying, it is used for transmission microscopy.

The particle sizes of EVs after ultracentrifugation were measured using a dynamic laser light scattering instrument (DynaPro NanoStar, Wyatt, USA). Here, 10 μL of EVs was taken to record the hydrodynamic diameter of the vesicles using this instrument, and results were exported after the data were analyzed using its own software. Concentration was measured using a nanoparticle tracking analyzer (Zeta View, Particle Metrix, Germany). First, the sample pool was washed with deionized water, and it was cleaned again after calibrating the instrument with 100 nm polyphenylene propylene microspheres. Then, 25 μL of EVs was taken and diluted 100 times with PBS. Finally, the diluted solution was loaded using a syringe.

After measuring concentration, different EV samples were mixed with SDS-PAGE sample loading buffer (5×), boiled at 100 °C for 10 min, and placed on ice for 5 min. Subsequently, 10 μg of the sample was added to the prepared 12.5% SDS-PAGE gel electrophoresis well. After running the gel at 100 V for 90 min, the SDS-PAGE gel was removed and transferred to the PVDF membrane. The PVDF membrane was blocked with 5% skimmed milk powder for 1.5 h (EPS 600, Tianneng, China) and incubated with primary antibodies CD63 (dilution ratio 1: 500) and TSG101 (dilution ratio, 1:1000) overnight in a 4 °C refrigerator shaker. Membranes were washed thrice with PBST for 5 min each, and then incubated for 1 h at room temperature with horseradish peroxidase-conjugated secondary antibodies (goat anti-mouse for EV proteins and human serum proteins and goat anti-rabbit immunoglobulin G for lipoprotein) at a dilution ratio of 1:5000. The membrane was washed thrice with PBST for 5 min each time. Finally, a chromogenic solution was added and developed in a chemiluminescence instrument (5200CE, Tianneng, China).

### Synthesis of SERS substrate AuNPs

Gold nanoparticles (AuNPs) were prepared by sodium citrate reduction 41, 42. Tetrachloroauric acid trihydrate was used as the gold source, and sodium citrate was used as the reducing agent. During the whole reaction, the glassware involved in the experiment was cleaned and soaked in aqua regia (HCl:HNO_3_ = 3:1) for 30 min to remove inorganic impurities. Then, it was immersed in piranha solution (H_2_SO_4_:H_2_O_2_ = 3:1) for 30 min to eliminate organic impurities. Finally, it was rinsed thoroughly with deionized water to prevent other impurities from interfering with the formation of colloidal microporous particles. The specific synthesis method is as follows:

200 mL of ultrapure water and 1.5 mL of 1% sodium citrate solution were added to a round-bottomed three-necked flask. After stirring and heating to boiling, 2.42 mL of 1% tetrachloroauric acid solution was added quickly, and the stirring and heating reaction was continued for 40 min. The color changed from light yellow to black, and finally changed to red until the color of the synthetic solution no longer changed. Stop heating and continue stirring for 1 h. When the synthetic solution was cooled to room temperature, a part of the gold nanosolution was centrifuged at 8000 rpm for 20 min and preconcentrated five times for SERS detection, and the remaining part was stored at 4°C for subsequent characterization and use.

Using the above method, by adjusting the ratio of ultrapure water, 1% sodium citrate solution and 1% tetrachloroauric acid solution, two other particle sizes of AuNPs were synthesized and stored at 4°C for subsequent experiments.

#### Characterization of AuNPs

The visible spectra of AuNPs were measured using an ultraviolet-visible spectrometer (Evolution-201, Thermo Fisher, USA) with a wavelength range of 400-700 nm. The AuNPs were analyzed using TEM (JEM-2100Plus, JEOL, Japan) at an accelerated voltage of 200 kV to determine their size and morphology. The preparation method of the TEM measurement sample is as follows: a drop of colloidal solution was diluted properly and added dropwise to a 400-mesh copper mesh covered with an amorphous carbon film, and the solvent was evaporated in air at room temperature.

#### SERS signal enhancement, uniformity, and repeatability

Enhancement factor (EF) is a key parameter for evaluating the performance of SERS substrates, which can quantify the SERS effect. The calculation formula is as follows 43, 44:


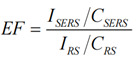
 (1)

where *C_SERS_* and *C_R_*_S_ are used to measure the Raman reporter concentrations of SERS and Raman spectroscopy (RS).* I_SERS_* and *I_RS_* are their signal intensities.

Rhodamine 6G is one of the most commonly used Raman reporter factors in SERS, and its single-molecule resonance SERS sensitivity has long been demonstrated 45. Therefore, in this experiment, R6G was used to determine its EF on AuNPs. The instruments, laser wavelength, laser power, objective lens, and integration time used were consistent. In the RS experiment, some weak peaks were observed in Raman signals at a high concentration (1×10^-2^ mol L^-1^) of R6G, with an *I_RS_* of 3299.83 at 1363 cm^-1^. In the SERS experiment, a lower concentration (1×10^-5^ mol L^-1^) of R6G was formulated to minimize the spectral signal that may be generated in the optical path outside of the SERS hotspot, with an *I_SERS_* of 34962.74 at 1363 cm^-1^.

To evaluate the uniformity of the SERS substrate and reproducibility of the enhanced Raman signal, the Raman signal of R6G with a concentration of 10^-5^ mol L^-1^ was measured at 24 different random positions of a single sample using the 44.06 nm AuNP substrate.

### SERS measurement

SERS data were measured using an alpha300R confocal Raman microscope (WITec, Germany). The laser wavelength was 633 nm, the laser power is 10 mW, and the integration time was 30 s. The incident laser beam was focused on the surface of the sample using a 10× objective with an aperture of 0.25. For the measurement of EV-AuNPs heterogeneous samples, a lower N.A. objective can provide a larger focal spot, as capturing broader regions is more important than achieving the finest resolution. At the same time, a lower N.A. objective can also improve the overall signal-to-noise ratio, especially when using a longer exposure time or when the Raman signal intensity is low. And by using a low N.A. objective, the light is less concentrated, which also reduces the risk of photodamage. The 3 µL EV solution was dropped into the concentrated gold nanosol (with a volume ratio of 1:1) for detection (the structure of the EVs was not destroyed). For the EV-AuNPs solution, a confocal Raman imaging microscope was used to measure one spectrum at a uniform position of each sample (avoiding potential agglomeration areas), and then the next spectrum was measured by changing the sample. Because EVs are normally small and scarce, the EV-AuNPs solution is heterogeneous, and the Raman fingerprint can be easily confused with any other residual lipoproteins or biological cargo presented after the isolation process. Moreover, collecting spectra with new samples each time can avoid the interference signals caused by laser-induced physical or chemical changes. Therefore, such measurement steps can ensure the accuracy, repeatability, and representativeness of Raman spectral data.

For cell-derived (A549, BEAS-2B, HEK, HeLa, and HepG2) exosome samples, 135 samples were prepared for each type of EV, resulting in 135 spectra. In order to evaluate the ability of this method to predict EVs in complex samples, we designed four types of mixed samples according to the method of Parlatan et al. 46. A549 and BEAS-2B cell-derived exosomes were mixed at the following concentration ratios: 99:1, 90:10, 75:25, and 50:50. 135 samples were prepared for each type of the mixed sample, resulting in 135 spectra.

For mice plasma-derived exosome samples, including 8 mice with lung cancer and 8 healthy mice, each sample was equally divided into 5 replicates, and 5 Raman spectra were measured in each replicate according to the above steps.

For human plasma-derived exosome samples, including 10 lung cancer patients and 8 healthy people, each sample were equally divided into 10 replicates, and 15 Raman spectra were measured in each replicate according to the above steps.

In the data analysis of each type of experiment, all spectral data (not every class of spectral data) were randomly divided into training, validation, and test sets at a ratio of 60 %, 10 %, and 30 %.

## Methods

### Preprocessing algorithms for Raman spectroscopy

The background (baseline) of Raman spectra can cause the signal of the analyte to be masked. Thus, an appropriate pretreatment is required to attenuate or even eliminate the background 23. The background removal algorithms reported in the literature include morphological manipulation 47, 48, polynomial regression 49, Bayesian learning 50, mixed models 51, 52, baseline estimation using genetic algorithms 53, and exogenous background correction 54. We chose morphologically weighted penalized partial least squares (MPLS) 48, which are accurate and effective and have been validated in various types of data including Raman spectra 55, 56. First, MPLS roughly estimated the contour of the background by mathematical morphological operation. Then, it used the penalty least squares method to refine the background contour, and finally subtracted the refined background from the original spectrum 48.

Reducing the noise level can improve the signal-to-noise ratio of a Raman sepctrum. Common methods include polynomial smoothing, discrete wavelet transform, and discrete Fourier transform (DFT) 57. DFT was used to smooth each spectrum to improve the signal-to-noise ratio because of its high fidelity. DFT first used a discrete Fourier transform to transform the Raman spectrum (time domain) to the frequency domain. Then a suitable window function was used to weaken or even eliminate the intensity at the frequency corresponding to the noise, while keeping the intensity at the frequency corresponding to the signal unchanged. Finally, a discrete inverse Fourier transform was used to transform it back to the time domain to obtain the high-fidelity, smoothed Raman spectrum. The formulas for the discrete Fourier transform and its inverse transformation are as follows:


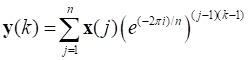
 (2)


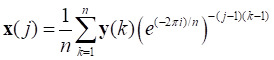
 (3)

where *n* is the length of the Raman spectral vector **x** and **y** is the frequency domain representation of **x**.

### Machine learning and deep learning

#### Support vector machines

The commonly used supervised learning algorithms in machine learning include discriminant analysis (DA), decision tree (DT), support vector machines (SVM), K-nearest neighbor (KNN), boosting tree (BT), and logistic regression (LR) 58. Among them, SVM is a very popular supervised learning method, which has the characteristics of flexibility, generalization ability, and suitability for few-shot learning 58, 59. First, the SVM embeds the data into a higher-dimensional space using a kernel function to generate a linear separation hyperplane. Then, to minimize the expected generalization loss, a maximal margin separator (a decision boundary with the maximum possible distance from the training point) is constructed to separate the data in the high-dimensional space 58. When mapped back to the original input space, the optimal linear separator constructed by SVM can correspond to the decision boundary between any wavy, nonlinear, positive and negative examples. With the exception of support vectors (those points closest to the separator that “block” the separation plane), the other data points in the SVM classifier have zero weights, and the support vectors are usually much less than the sample. Therefore, the SVM is a nonparametric method, which also gains some advantages of the parameterization.

The SVM algorithm divides the samples into positive classes (*y* = 1) and negative classes (*y* = -1), optimizes the target using the Lagrange multiplier method, introduces the coefficients *α*_1_,…, *α_n_*, and finds the optimal solution by solving the following formula:


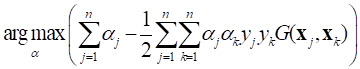
 (4)

The constraint Σ*α_j_ y_j_* = 0, box constraint ≥ *α_j_* ≥ 0 (which can be relaxed to *α_j_* ≥ 0 for linearly separable classes), and Karush-Kuhn-Tucker complementarity must be satisfied. **x***_j_* and **x***_k_* are the measurement data vectors of the *j*th and *k*th rows (samples) in data matrix **X**. *G*(**x***_k_*, **x***_j_*) are the elements in the Gram matrix, and different kernel functions correspond to different Gram matrices:


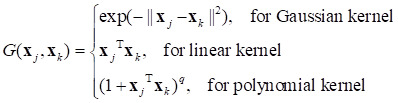
 (5)

The resulting score function is the following:


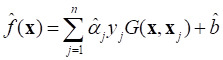
 (6)

where “ˆ” denotes the estimated value, and b is the bias. The SVM algorithm uses 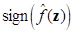
to classify the new sample data **z**.

#### Convolutional neural network

Deep learning, often categorized as a distinct branch of machine learning, is inspired by neural pathways in the human brain and typically uses neural network architectures to learn to perform classification or regression tasks directly from images, text, or sound, and the term “depth” usually refers to the number of hidden layers in a neural network 60. Common techniques for deep learning include CNN, recurrent neural networks (RNN), long short-term memory (LSTM), and Transformer neural networks. Deep learning models are trained using large amounts of labeled data, and automatically learn features directly from the data without manually extracting features. Models often continue to improve as the amount of data increases, but deep learning requires a lot of computing power.

CNN is one of the most popular deep learning networks that automatically learn relevant features through input data and have high classification accuracy 60, 61. The CNN consists of an input layer, a number of hidden layers in the middle, and an output layer, which transmits a spectrum or an image forward into the network, and each layer of the network learns to detect different features, uses the output as the input of the next layer, and finally, outputs the classification result 58, 60, 62. The feature learning layer automatically learns features, among which the convolution, pooling, and rectified linear unit (ReLU) layers are the most common. The convolution layer applies a set of convolutional filters to the input spectrum or image, and each filter activates specific features in the data. The ReLU layer maps the negative value to zero while keeping the positive value unchanged through the function ReLU(*x*) = max (0, *x*) to improve the speed of training. Only the activated features are passed to the next layer, which is why the ReLU layer is also called the activation layer. The pooling layer performs nonlinear downsampling to simplify the output.

If **W**^(1)^ and **W**^(2)^ represent the weight matrices of the first and second layers and **g**^(1)^ and **g**^(2)^ represent the activation functions of the first and second layers respectively, then the two-layer network can be expressed as follows:


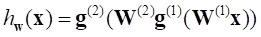
 (7)

If the prediction of the output of the network is 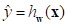
and its true value is *y*, the loss function can be expressed as follows:


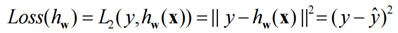
 (8)

In the output layer, the output value is expressed as a probability to find **w** that maximizes the probability of the observed data:


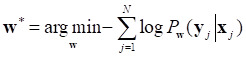
 (9)

Here, we use the cross-entropy *H*(*P*,*Q*) as the loss function to minimize the cross-entropy by adjusting **w**, where *P* is the true distribution of the training examples, and *Q* is the hypothetical prediction *P***_w_** (*y* | *x*). The cross-entropy is defined as follows:


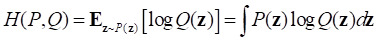
 (10)

After the feature learning layer, the CNN architecture outputs the classification results. The fully connected layer outputs a *d*-dimensional vector (*d* is the number of classes in the model), which gives the probability that the samples will be assigned to each class. The final softmax layer gives the final classification output 58, 60. For a *d*-dimensional input vector **in** = [*in*_1_, . . ., *in_d_*], a vector of the same length will be outputted, and its *k*th element can be expressed as follows:


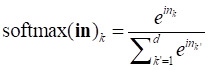
 (11)

The above data analysis uses MATLAB R2021a (MathWorks, USA) software, running on an ordinary computer (Intel Core TM i3-8100 CPU @ 3.60 GHz, 8 GB RAM).

## Results and Discussion

### Characterization of SERS substrate AuNPs

**Figure 2:**
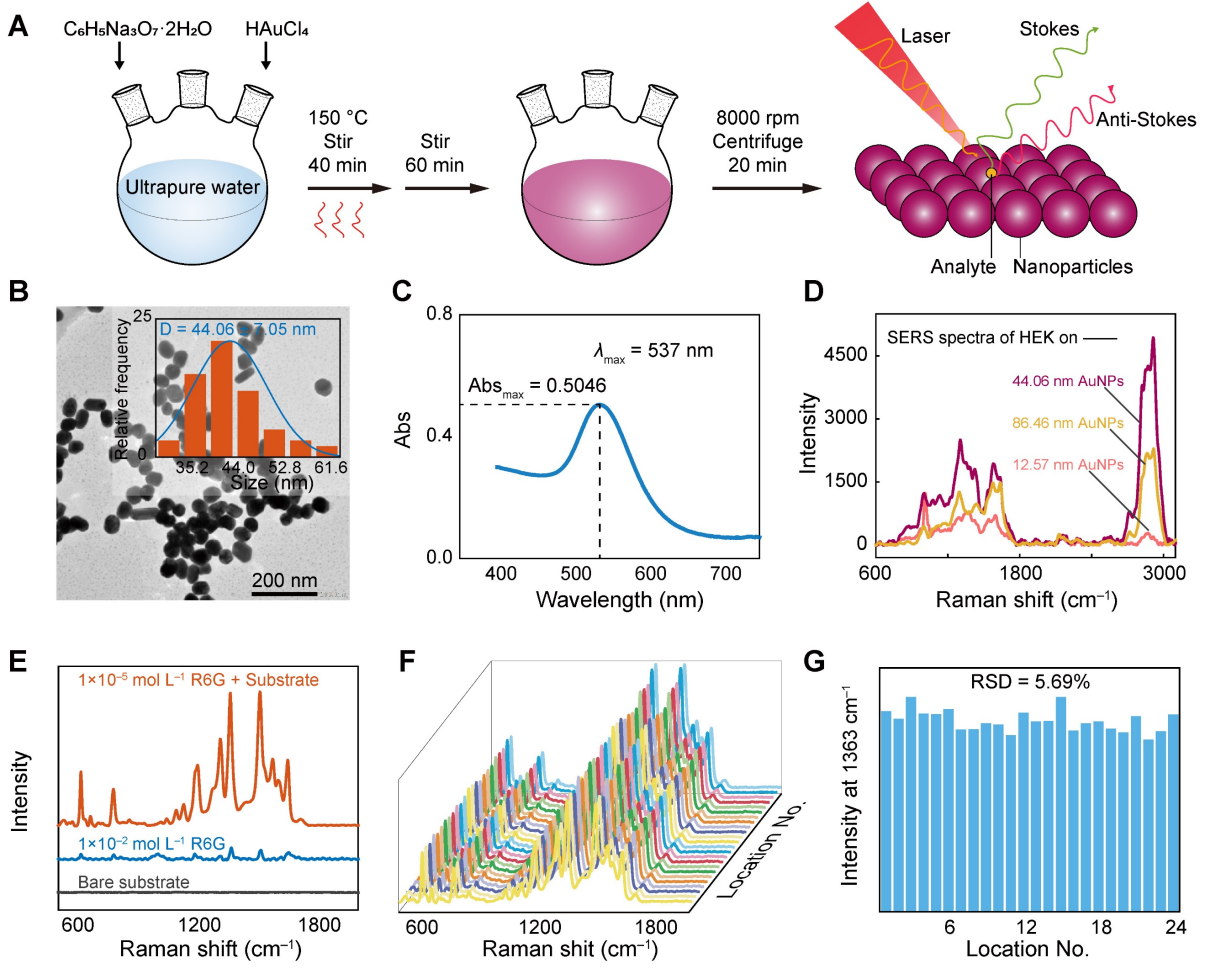
Characterization and performance testing of AuNPs. (A) Schematic of AuNPs synthesis and SERS detection. (B) TEM and (C) ultraviolet-visible absorption spectra of the AuNPs. (D) Enhancement of exosomes by three different sizes of AuNPs (taking HEK as an example). (E) Raman spectra of R6G (1 × 10^-2^ M) and SERS spectra measured by dropping R6G (1 × 10^-5^ M) in AuNPs. (F) SERS spectra measured at 24 different locations on the substrate for 10^-5^ M R6G. (G) The band intensities and their relative standard deviations of the SERS spectra measured at 1363 cm^-1^ and at the 24 locations above.

To achieve the highly sensitive detection of low-signal cell-derived EVs by the label-free SERS method, AuNPs were prepared as SERS substrates to enhance the Raman signal and characterized. The size, shape, and degree of aggregation of nanoparticles have a significant effect on SERS enhancement 63, 64. Therefore, we prepared AuNPs of three different sizes, namely, small, medium, and large (Figure 2A), with particle sizes of 12.57, 44.06, and 86.46 nm, respectively (Figure 2B and Figure S3A-B). Owing to the surface plasmon resonance characteristics of gold, it has scattering and absorption effects on visible light, and as the particle size increases, the scattering and absorption wavelengths are red-shifted and the absorption peaks become widened 65, 66. The maximum absorption wavelengths are observed from the visible spectrum at 520, 537, and 545 nm, respectively (Figure 2C and Figure S3C-D). The relationship between the maximum absorption wavelength and the particle diameter indicates that the synthesized solution is indeed AuNPs. Figure 2D shows the enhancement effect of three different sizes of AuNPs on EVs. For larger particle sizes, various hotspot configurations may be generated 67, and the larger particle size AuNPs have less electrostatic repulsion between each other and are more likely to aggregate in solution 68. As shown in the figure, the enhancement effect of the 44.06 nm AuNPs is stronger than that of the 12.57 nm ones. However, as the particle size increases further, the analyte signal does not increase with the particle size, which may be due to the fact that as the nanoparticle size increases, the convex shape of the surface is no longer a complex polyhedral, but becomes flatter, resulting in less light absorbed by the particles and less inelastic scattering occurring on the surface, ultimately reducing the surface electromagnetic field and overall SERS intensity 64, 69. As shown in the figure, the enhancement effect of 86.46 nm AuNPs is weaker than that of 44.06 nm. In addition, according to Mie's theory, for AuNPs below 20 nm, extinction is almost entirely due to absorption. With an increase in particle size, the optical cross-section is larger and the extinction ratio of scattering increases. When the particle size is increased to 80 nm, the degree of extinction of absorption and scattering is similar 70, 71. Therefore, we chose medium-sized (44.06 nm) AuNPs in this experiment.

We first evaluated the SERS signal on the bare substrate (Figure 2E), which had few signal peaks. High uniformity and reproducibility are two essential indicators of high-quality SERS substrate. The uniformity and reproducibility of the AuNP substrate were evaluated under the same experimental conditions using SERS spectra of reporter factor R6G at a concentration of 10^-5^ M, as shown in Figure 2E-G. In the absence of AuNPs, only a few weak Raman peaks were observed. However, in the SERS spectrum of R6G with AuNPs, the intensity of many dominant vibration bands increased significantly, with an EF of approximately 1.04 × 10^4^ for R6G detection (Figure 2E). Figure 2F shows SERS spectra measured at 24 different locations on the substrate for 10^-5^ M R6G. To obtain statistically significant results, the signal intensity at 1363 cm^-1^ was plotted as a histogram (Figure 2G), and the peak intensities were very consistent across the 24 random positions, with a relative standard deviation (RSD) of 5.69%. This small RSD shows that although AuNPs have an uneven arrangement of hotspots, it can provide an orderly electromagnetic field distribution, resulting in a relatively uniform signal with high uniformity and reproducibility.

### Characterization of EVs

**Figure 3:**
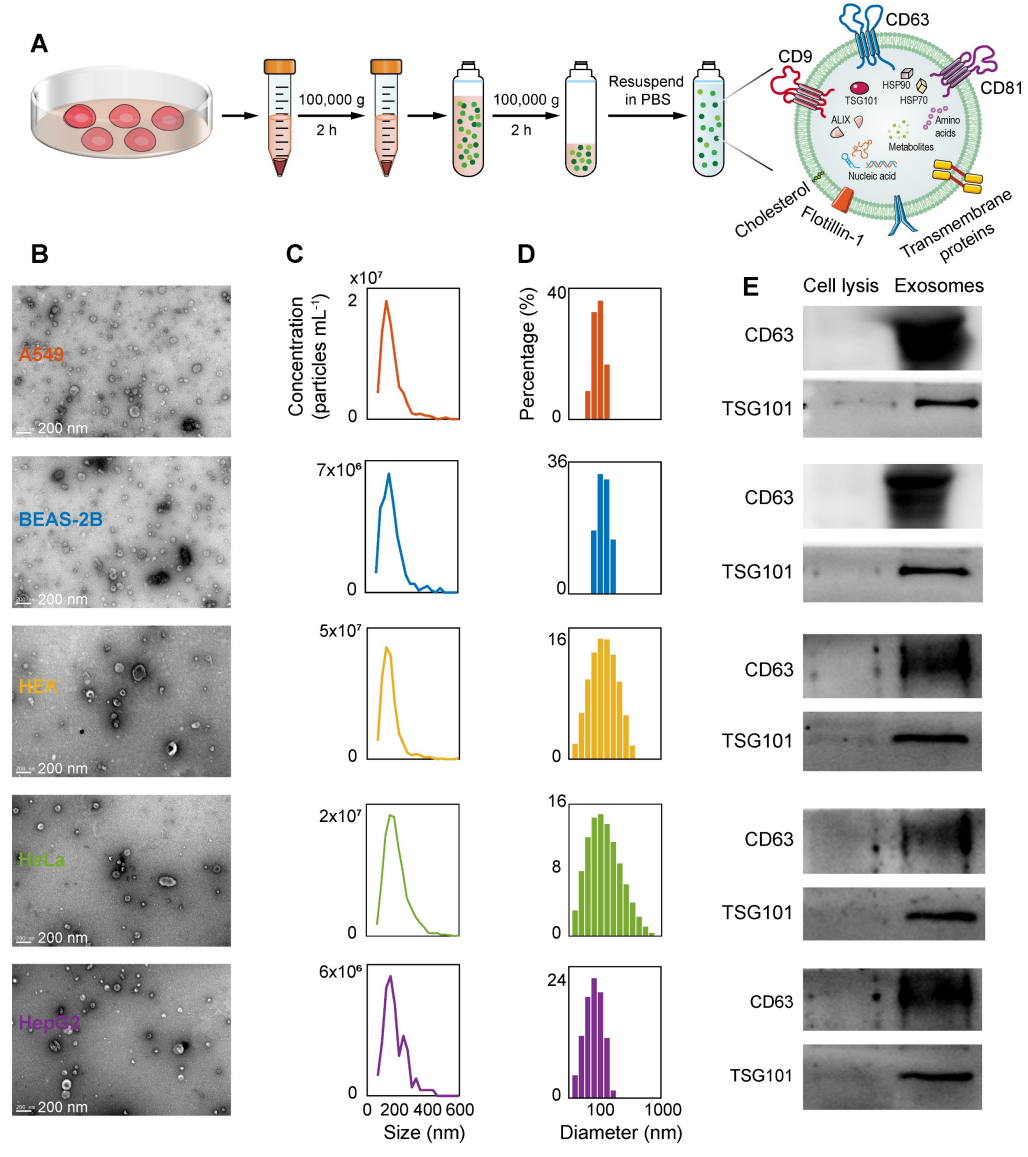
Isolation and characterization of EVs. (A) The process diagram of the ultracentrifugation separation of the external vesicles. (B) TEM images characterizing the morphology of the isolated vesicles (scale bar: 200 nm). (C) NTA results for five EVs. (D) DLS particle size distribution of five EVs. (E) WB results of EV markers CD63 and TSG101.

The ultracentrifugation extraction of EVs from the A549, BEAS-2B, HEK, HeLa, and HepG2 cell lines and the characterization results of TEM, NTA, dynamic light scattering (DLS), and WB are shown in Figure 3. Figure S4 and Figure S5 show the TEM and NTA results of mouse and human plasma-derived exosomes, respectively. Figure 3A illustrates the extraction of exosomes using ultracentrifugation. The sizes of these EVs vary from different samples, as confirmed by the TEM characterization results (Figure 3B, Figure S4A, and Figure S5A), which show that the diameters of these types of vesicles are mainly distributed between 100 and 200 nm. The TEM images also show that these vesicles have a typical double-membrane morphology, and no obvious protein aggregates that could interfere with the signal are found in the images. The concentration distributions of the various types of EVs at different sizes were obtained using the NTA technique (Figure 3C, Figure S4B, and Figure S5B), and the total concentrations of EVs were calculated to be 8.7 × 10^9^, 3.2 × 10^9^, 1.7 × 10^10^, 9.4 × 10^9^, and 2.9 × 10^9^ particles mL^-1^, respectively. The concentrations of plasma-derived exosomes in lung cancer and healthy mice were 3.0 × 10^9^ and 1.8 × 10^9^ particles mL^-1^. The concentrations of plasma-derived exosomes in lung cancer patients and healthy people were 3.1 × 10^10^ and 1.4 × 10^11^ particles mL^-1^. Then, DLS analysis was performed to further examine the size distribution of the various EVs (Figure 3D), with weighted average diameters of 126.9, 155.4, 172.8, 191.5, and 109.8 nm, respectively. As previously shown by the differences in particle size distribution between EVs in normal and cancerous cell lines 72. In addition, for a more in-depth assessment of EVs, we performed a western blot test on the membrane proteins of each EV (Figure 3E). The WB results showed that the common EV markers CD63 and TSG101 were expressed in these EVs, despite the different sizes of the vesicles. These results fully confirm the successful isolation and purification of EVs from five cell lines.

### Spectral preprocessing and peak assignment analysis

**Figure 4:**
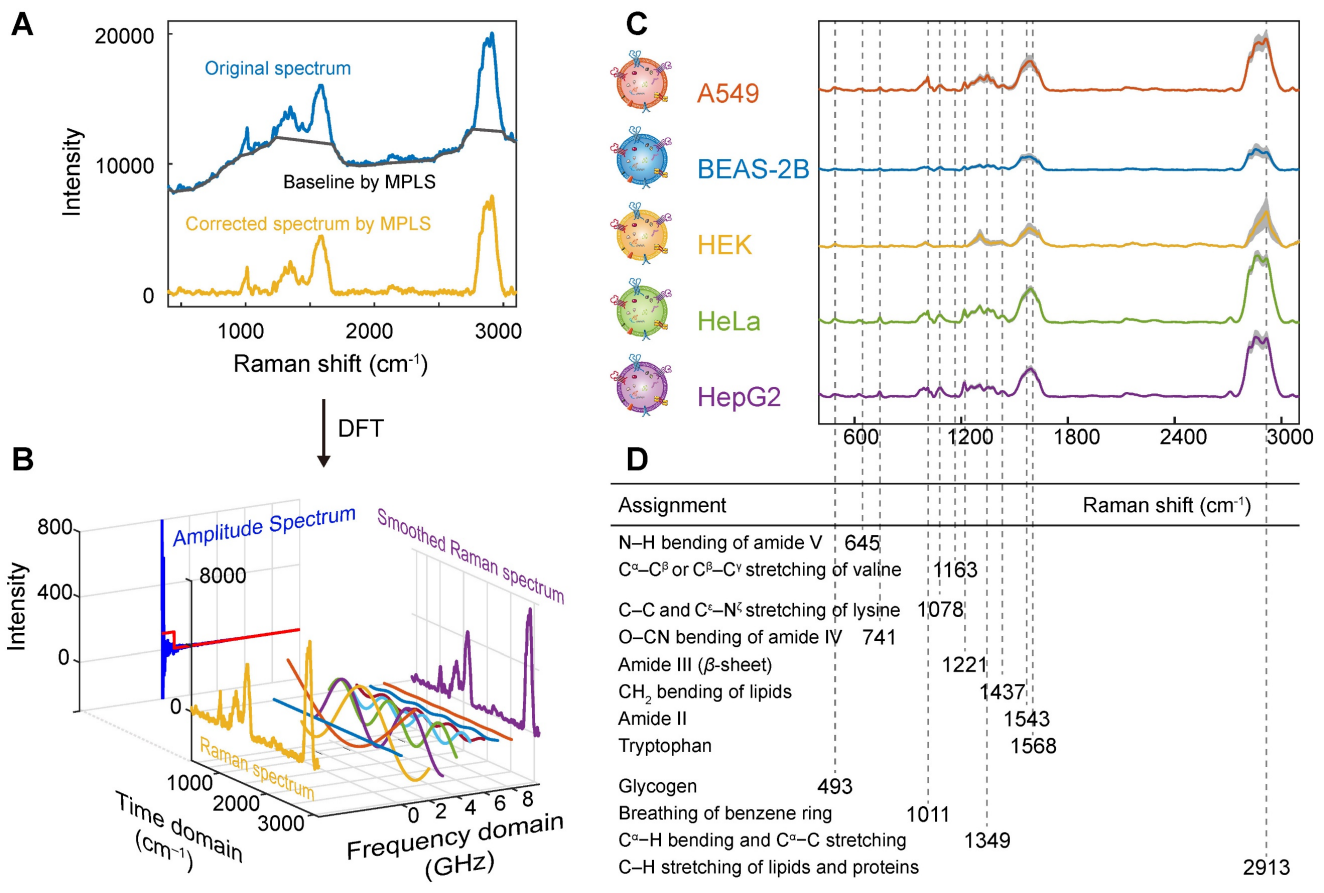
Spectral preprocessing and peak assignment analysis. (A) MPLS-based background removal. (B)Smoothing using DFT. (C) Raman spectra after pretreatment of A549, BEAS-2B, HEK, HeLa, and HepG2. (D) Peak attribution results. Spectra from the first sample of A549 are selected in (A) and (B) to show the spectral preprocessing process.

Background interference is also present in the Raman spectra of EVs after the removal of cosmic rays (Figure 4A). In this regard, the MPLS algorithm is used to remove the background of each spectrum. The algorithm parameters are as follows: the width for structuring elements in morphological operation is set to 75, the penality parameter is set to 10, the flat region proportion at both the start and end is set to 1/2000, and the order of the difference in penalties is set to 1. After subtracting the fitted background (gray curve), a normal Raman spectrum (golden curve) with a smooth baseline is obtained, which emerges with clearer Raman bands.

For the spectrum after background removal, discrete Fourier transform is used for smoothing, as shown in Figure 4B. First, the spectrum of the time domain (yellow) is represented by a Fourier series, and the amplitude spectrum (blue) of the frequency domain is obtained by transforming. Then, use a window function (red) to eliminate the amplitude of the noise-containing frequencies. Finally, the inverse discrete Fourier transform is used to transform the frequency domain signal back to the spectrum of the time domain, that is, the smoothed spectrum (purple).

The spectra and band assignment analyses of pretreated EVs from A549, BEAS-2B, HEK, HeLa, and HepG2 cells are shown in Figures 4C and D. As shown in the figure, the Raman spectra of EVs from different cell origins, including cancer cells and normal cells, are highly similar, with a high degree of Raman spectra at 493 cm^-1^ (glycogen) 73, 74, 741 cm^-1^ (O-CN bending of amide IV) 75, 1011 cm^-1^ (breathing of benzene ring) 76, 1078 cm^-1^ (C-C and C^ε^-N^ζ^ stretching of lysine) 77, 1221 cm^-1^ (amide III (β-sheet)) 78, 1349 cm^-1^ (C^α^-H bending and C^α^-C stretching) 79, 1437 cm^-1^ (CH_2_ bending of lipids) 80, and 2913 cm^-1^ (C-H stretching of lipids and proteins) 81. The presence of these characteristic bands indicates that the isolated EVs contain components such as lipids and proteins. In addition, the broad and strong signal commonly seen around 1598 cm^-1^ may be derived from citrate molecules on the surface of AuNPs. These peak assignment analyses showed that their components were similar. However, there are subtle differences in the peak position and intensity in bands around 741, 1011, 1078, 1221 and 1349 cm^-1^ of different cell-derived EVs, indicating that their composition (such as lipids and proteins) is different. Among them, at 1349 and 1437 cm^-1^, the SERS intensity of cancer cell-derived EVs was higher than that of normal cell-derived EVs because of the large amount of collagen in cancer cells as an essential source of energy for growth, and the abnormal DNA or RNA metabolism in cancer cells, resulting in higher levels of collagen and adenine than normal cells 82-84.

For example, the SERS spectra of lung cancer cell A549 and normal lung cell BEAS-2B derived EVs are highly similar, but they also show some differences. The SERS intensity of BEAS-2B cell-derived EVs at 1078 and 1221 cm^-1^ was stronger than that of A549 cell-derived EVs, because of the intense metabolism of cancer cells leading to lower amino acid levels 85. It can also be attributed to the difference in the content of ergothioneine (ET), which is due to the role of ergothioneine in antitumor processes by inducing reactive oxygen species (ROS)-mediated cytotoxicity so that cancer cells have lower ergothioneine levels than normal cells 86, 87. When normal lung cell BEAS-2B was transformed into tumor cell A549, the intensity of some protein-related bands (e.g., 493, 1011, 1349, and 2913 cm^-1^) in EVs increased. Some new bands (e.g., 645 and 1163 cm^-1^) related to lipids, proteins, and DNA appeared. Although A549 cell-derived EVs show some characteristic peaks, not every cancer cell-derived EVs have these characteristic peaks, and the enhancement of these characteristic peaks may be due to the randomness of the interaction and bonding between AuNPs and EVs. The subtle differences in the bands observed in SERS and their intensities suggest the potential to distinguish EVs using the Raman bands of lipids and proteins, but these differences are not significant enough to be directly classified. Therefore, we intend to introduce a machine learning algorithm to construct a reliable classification model based on the complete SERS spectrum.

### Classification of A549- and BEAS-2B-derived EVs

#### Comparison and optimization of machine learning algorithms

Algorithm selection often depends on the characteristics of the specific datasets, which will have a crucial impact on the results. Here, we compare the classical machine learning (ML) algorithms, including DA, DT, SVM, KNN, and BT. After the spectra were preprocessed by MPLS and DFT, we use the same training set and test set to evaluate the classification performance and CVloss of each algorithm. The overall accuracy and CVloss of the above algorithms are listed in Table 1, and these data show that SVM is superior to other traditional ML algorithms. SVM often gives better prediction results for small sample data 88, especially for more accurate models built through hyperparameter optimization, which is more flexible. Therefore, we choose the SVM algorithm to construct the classification model based on the SERS data of EVs.

#### Using SVM to train machine learning models

**Figure 5:**
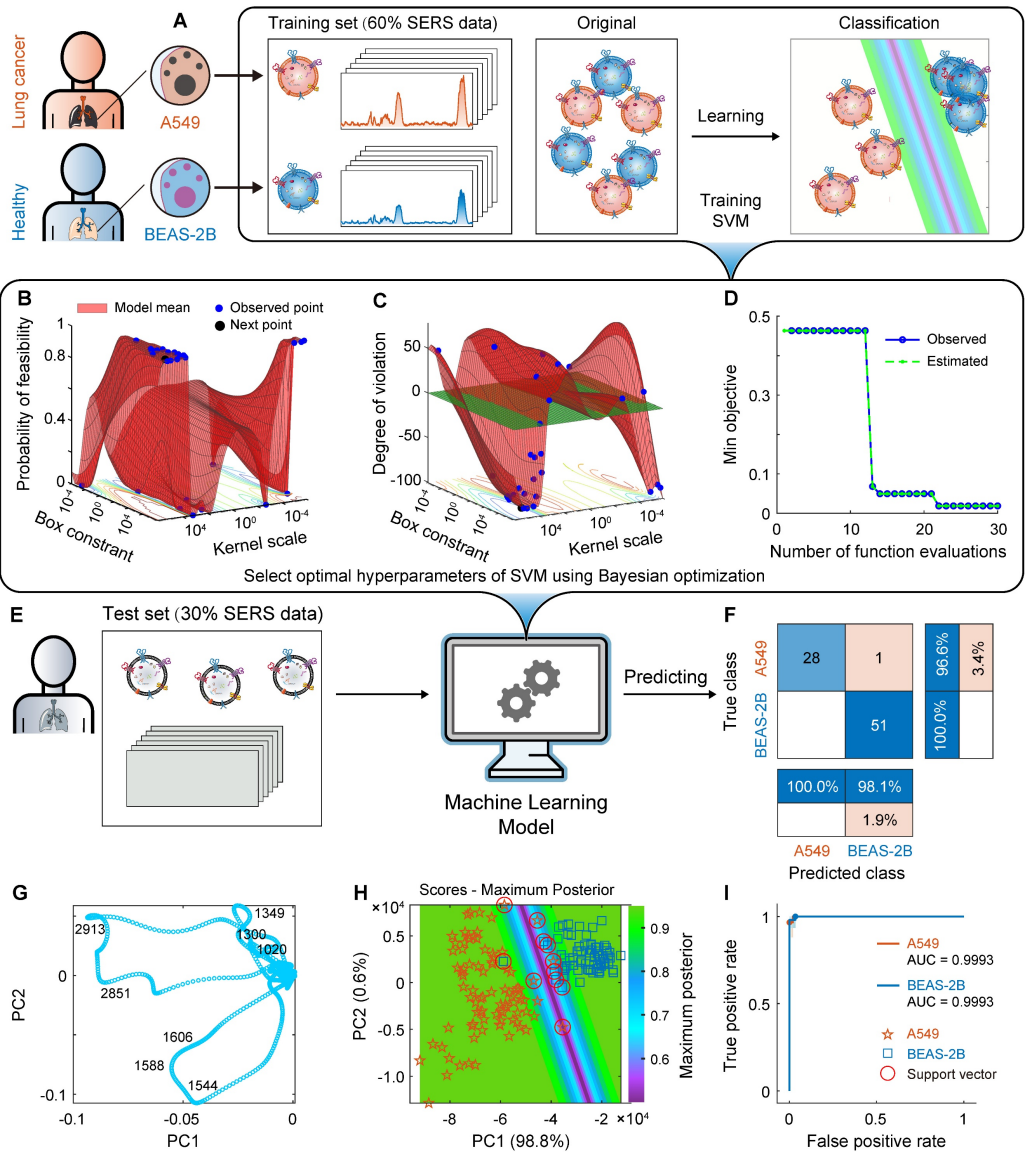
Machine learning (ML) model construction and prediction of A549 and BEAS-2B cell-derived EVs. (A) Schematic for constructing a ML classification model based on EV SERS spectra. (B) and (C) use Bayesian optimization to optimize the parameters of the SVM algorithm. (D) Convergence plot of the SVM algorithm. (E) Independent test set. (F) Confusion matrix using the SVM model for independent test sets. (G) Loading plot of PCA. (H) Maximum posterior probability plot. (I) ROC curves and AUC values for the independent test set.

The process schematic, parameter optimization and prediction results of using SVM to construct ML models for A549 and BEAS-2B cell-derived EVs are shown in Figure 5. First, to explore the distance between cancer cell EVs and normal cell EVs in the high-dimensional space, we used PCA for exploratory data analysis (the spectra were preprocessed with MPLS and DFT). The first principal component PC1 captured most of the variance (98.8%), and the high variance ensured the representativeness of the PCA model. The second principal component PC2 interpreted 0.6% of the information. As shown in Figure 5H, the EVs SERS spectra from these two types of cells are well clustered. As mentioned above, there are some differences in the SERS spectra of EVs derived from lung cancer cells and normal lung cells. In addition, the loading map for PCA (Figure 5G) shows that the four SERS bands around 1020, 1300, 1349, 1544, 1588, 1606, 2851, and 2913 cm^-1^ are important variables that have essential contributions to clustering. These SERS bands are related to lipids and proteins, indicating that lipids and proteins are characteristic components of EVs.

However, pattern recognition based on small biological datasets (low sample size) is challenging because of the complexity and heterogeneity of EVs, and models still need to be optimized to achieve the required sensitivity and accuracy. As mentioned above, after using PCA to reduce the data to two dimensions, SVM was used to construct a ML classification model for A549 and BEAS-2B cell-derived EVs, in which the linear kernel function was selected, the sequence minimum optimization was selected as the solver, and the acquisition function was “expected-improvement-per-second-plus.” To build a satisfactory classification model, we optimized the hyperparameters of the SVM algorithm 89, which is also called hyperparameter search. It improves not only the performance of the training process but also the accuracy of the algorithm. Various algorithms, such as grid search, gradient-based optimization, and Bayesian optimization, can be used for hyperparameter search. Here, we used Bayesian optimization (Figure 5B-D), where the optimum box constraint is 952433.64 and the optimum kernel scale is 158491.21.

On the basis of the above hyperparameter optimization results, we used the optimized SVM to construct a classification model for pattern recognition. All spectra have been preprocessed using MPLS and DFT. In the first step, on the basis of the SERS spectral matrix **X**training and class label vector **y** of the training set, the SVM algorithm is used to train the ML model. The algorithm parameters are as follows: the kernel function is linear, the box constraint is 952,433.64, and the kernel scale is 158,491.21. The training time of the SVM model is only 6.8905 s, and the prediction speed is approximately 220 observations per second. The CVloss of the model is 3.7%. In the second step, the SERS spectral matrix **X**test of the independent test set is brought into the SVM classifier to predict the class attribution of each sample. The confusion matrix and the receiver operating characteristic curve (ROC) are shown in Figures 5F and I. The confusion matrix results of the independent test set showed that the percentages of correctly and incorrectly classified observations for the true class of A549 are 96.6% and 3.4%, respectively; the percentages of correctly classified observations for the true class of BEAS-2B is 100%. The overall accuracy of the SVM model for the test set reached 98.7%. For 80 independent test samples, only one A549 sample was incorrectly predicted as BEAS-2B. The AUC of ROCs for both A549 and BEAS-2B cell-derived EVs was 0.9993. The maximum posterior probability plot (Figure 5H) intuitively shows the maximum margin separator of the SVM and its satisfactory classification results.

The above results show that the SVM classification model based on hyperparameter optimization has a satisfactory overall accuracy for EVs secreted by tumor cell lines and normal cell lines (A549 and BEAS-2B), and the overall accuracy (98.7%) is further improved than the value (94.4%) reported in the literature 34. The first reason may be that when performing SERS detection, the combination of the sample and the substrate was changed. Instead of the commonly used and more expensive water immersion objective, a capillary-based method was employed for analyzing EV samples in solution without a drying step, which is more uniform than the silicon wafer. This approach enables more efficient SERS signal enhancement by preserving the EVs in their liquid state within the capillary, thus improving measurement efficiency and signal quality. The second reason may be that bayesian optimization was used to optimize the hyperparameters of the SVM algorithm and train a model with strong classification ability. The improvement of the above two aspects is the innovation of the proposed method.

It is worth mentioning that SVM has satisfactory modeling ability for small samples. Our SVM classification model based on small samples (the number of samples per class is 135) has achieved satisfactory overall accuracy, which is valuable in the case of small sample sizes in practical applications. Therefore, on the basis of the small samples, we used label-free SERS technology combined with ML algorithm SVM to accurately classify healthy lung cells and their cancerous cell-derived EVs. In order to evaluate its application potential, the predictive ability of this method needs to be verified in more complex systems.

### Classification of EVs in five different cells

#### SVM classification model

We have trained an SVM classification model to identify normal lung cells and lung cancer cells in the previous article, but in practical applications, we may encounter other cells or other cancer cell samples, such as HEK, HeLa, and HepG2, which is essential to distinguish them. In this regard, we further investigated the classification model of EVs secreted by five cell types (A549, BEAS-2B, HEK, HeLa, and HepG2). On the basis of the peak assignment analysis above, although their SERS spectra are highly similar, there are some differences in some bands. If the established classification model can accurately identify each EV in the presence of various cell-derived EVs and can obtain high classification accuracy, then it will have better clinical application prospects and value. For this, we continued to train a ML classification model using SVM (the results are shown in Figure S6 of the supporting material). The confusion matrix of the independent test set (Figure S6A) showed that the true positive rate (recall) of A549, BEAS-2B, HEK, HeLa, and HepG2 is 100%, 98.0%, 100%, 100%, and 100%, respectively, and the AUC values of ROC is 1, 0.9945, 1, 1, and 1 (Figure S6B). The overall accuracy of the SVM model for the test set is 99.5%, and the CVloss of the SVM model is 0%. For BEAS-2B, although the recall is slightly lower than the previous results, it is still sufficient. These results showed that the SVM model still had satisfactory classification accuracy for EVs secreted by five different cell lines (A549, BEAS-2B, HEK, HeLa, and HepG2).

#### CNN classification model

**Figure 6:**
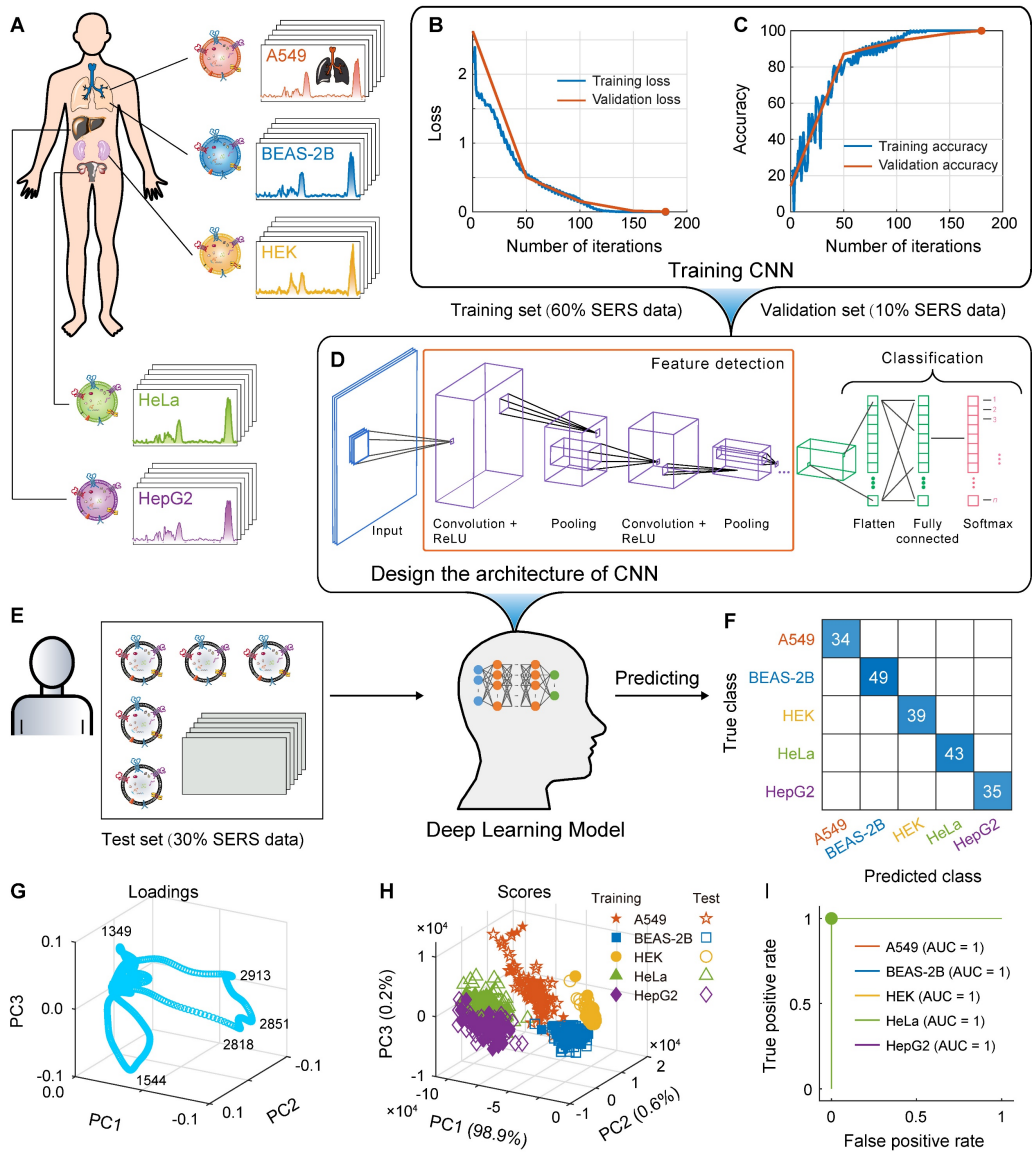
Construction and prediction of deep learning models of five cell-derived EVs. (A) Schematic of five cell sources. (B) Loss function (cross-entropy loss) curves of the training set and validation set. (C) Accuracy curves of the training set and validation set. (D) Schematic of the CNN model architecture. (E) Independent test set. (F) Confusion matrix using the CNN model for independent test sets. (G) Loading plot of PCA. (H) Score plot of PCA. (I) ROC curves and AUC values of the independent test set.

Similarly, we performed an exploratory analysis using PCA, selecting the first three principal components (total variance explained = 99.7%) to draw the loading plot and score plot in three-dimensional space, as shown in Figures 6G and H, respectively. The EVs of A549, BEAS-2B, HEK, HeLa, and HepG2 cells were clustered into five classes to some extent, and the bands at 1349, 1544, 2818, 2851, and 2913 cm^-1^ were the significant variables that contributed to clustering. However, there was overlap in the clustering of different cell-derived EVs, indicating that the five cell-derived EVs could not be well distinguished by PCA alone.

Further, as shown in Figure 6, we use CNN in deep learning to design and train classification model for the above five cell-derived EVs. As shown in Figure 6D, we designed a network architecture for CNN, in which convolution, ReLU, and pooling layers were used to automatically learn features from the SERS spectrum, and then the fully connected layer and softmax layer are used to output the probability and final classification results of the samples being classified into each class.

We used the designed CNN network architecture to train the deep learning classification model. The SERS spectra have been preprocessed using MPLS and DFT. In the first step, the CNN classification model was trained based on the SERS spectral matrix Xtraining and class label vector y of the training set, in which the solver was selected as adaptive moment estimation (Adam), the loss function was selected as cross-entropy loss, the maximum number of training rounds was set to 60, and the initial learn rate was set to 0.01. The loss function and accuracy curves of the training and validation sets are shown in Figures 6B and C, and the algorithm converged after the 180th iteration. In the second step, the SERS spectral matrix Xtest of the independent test set was introduced into the CNN deep learning model to predict the class attribution of each sample. The confusion matrix and ROC are shown in Figure 6F and I. The confusion matrix of the independent test set showed that the CNN model had a recall of 100% for A549, BEAS-2B, HEK, HeLa, and HepG2, respectively, achieving accurate classification, and the AUC of the ROC curve for all five types of samples is 1. The CVloss of the CNN model is 0.4%. These results indicate that the designed and trained the CNN deep learning model using the label-free SERS technology can accurately classify these five types of cell-derived EVs.

Compared with the classical SVM machine learning model, the CNN deep learning model is better at training more labeled data. The model often continues to improve with an increase in data volume, but requires more computing power. When choosing between machine learning and deep learning, one should consider whether there is a large amount of labeled data and high computing power. When there is less labeled data, it is more appropriate to use machine learning algorithms, especially SVM, as long as the overall accuracy meets the application requirements. When there is a large amount of labeled data and high computing power, more complex CNN deep learning models can be trained to obtain continuously improved classification results.

### Classification of the mixed samples of A549 and BEAS-2B cell-derived exosomes

It is also important to evaluate the predictive ability of the proposed method for EVs in complex mixed samples. In this regard, we studied the construction of a classification model for mixed samples of four different ratios (99:01,90:10,75:25, and 50:50) of A549 and BEAS-2B cell-derived exosomes, each of which has 150 spectra and a total of 600 spectra.

**Figure 7:**
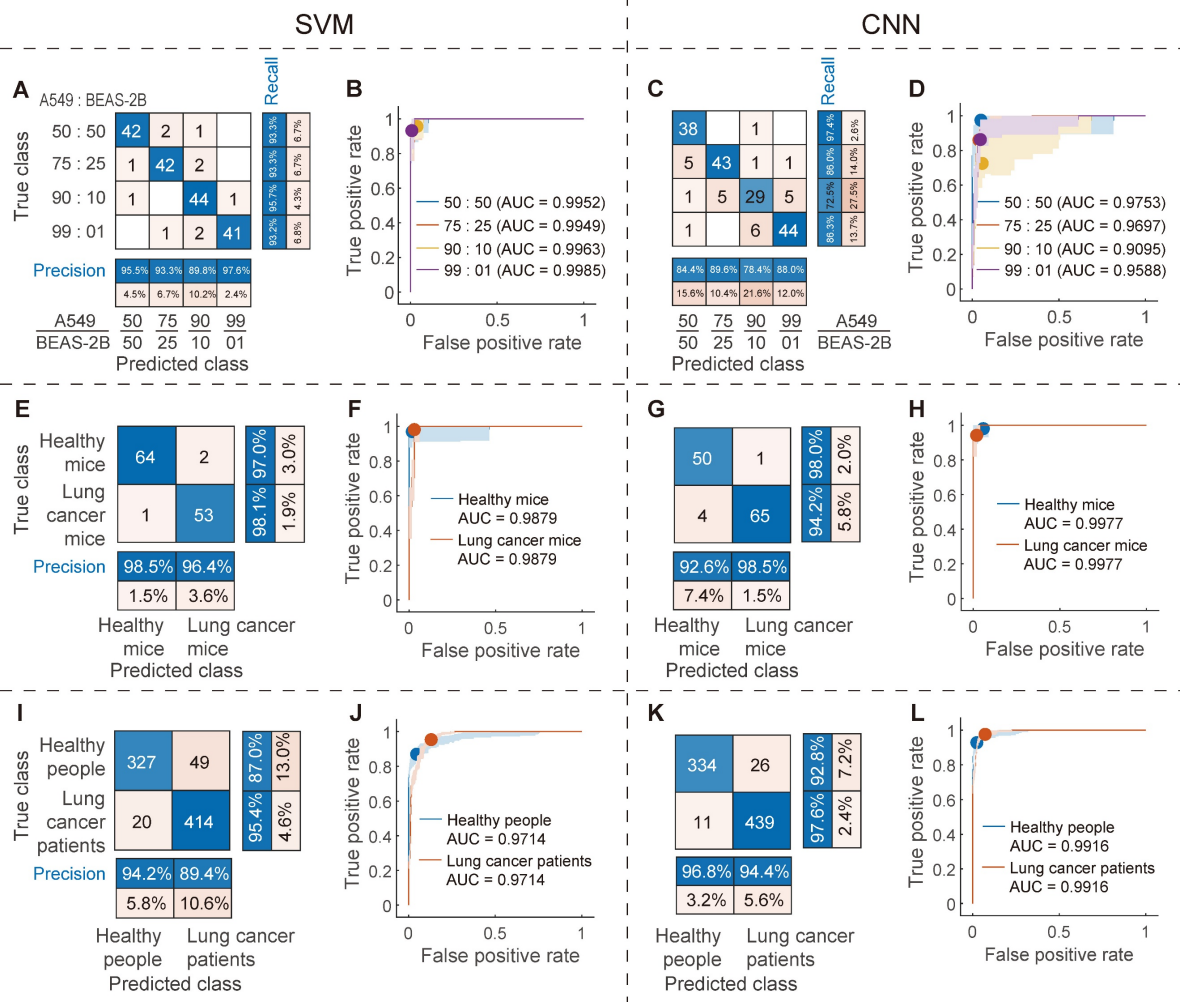
The classification results of the SVM and CNN models for the exosome mixed samples, animal samples and clinical samples, respectively. Confusion matrix (A) and ROC curve (B) of SVM and confusion matrix (C) and ROC curve (D) of CNN for independent test set of the mixed samples of A549 and BEAS-2B cell-derived exosomes. Confusion matrix (E) and ROC curve (F) of SVM and confusion matrix (G) and ROC curve (H) of CNN for independent test set of the plasma-derived exosome samples from lung cancer and healthy mice. Confusion matrix (I) and ROC curve (J) of SVM and confusion matrix (K) and ROC curve (L) of CNN for independent test set of the plasma-derived exosome samples from lung cancer patients and healthy people.

For the machine learning classification model based on SVM with hyperparameter optimization, the CVloss is 8.3%; for the independent test set (180 spectra, 30% of the total data), the recall of the four mixed samples was 93.2%, 95.7%, 93.3%, and 93.3%, respectively (Figure 7A), AUC was 0.9985, 0.9963, 0.9949, and 0.9952, respectively (Figure 7B), and the overall accuracy was 93.9%. For the deep learning classification model based on CNN with hyperparameter optimization, the CVloss is 17.8%; for the same independent test set, the recall of the four mixed samples was 86.3%, 72.5%, 86.0%, and 97.4%, respectively (Figure 7C), and the AUC was 0.9588, 0.9095, 0.9697, and 0.9753, respectively (Figure 7D). The overall accuracy was 85.6%, which was lower than that of the SVM model. For the SVM machine learning classification model, the overall accuracy of the mixed samples of these cell-derived exosomes was reduced by 4.8 percentage points compared to the 98.7% overall accuracy of the individual cell-derived exosome samples, which was expected. This is because the mixed samples mixed various types of features, which will increase the difficulty of classification. However, the overall accuracy is still satisfactory and is comparable to the values reported in the literature in other systems. These results show that the proposed SVM and CNN models still have satisfactory classification accuracy in the mixed samples of A549 and BEAS-2B cell-derived exosomes.

### Classification of the plasma-derived exosome samples from lung cancer and healthy mice

In order to further evaluate the application potential of the proposed method, a mice model of lung cancer was constructed, with a total of 400 spectra of 200 spectra in each class. For the machine learning classification model constructed by SVM, the CVloss is 2.5%; for the independent test set (120 spectra, 30% of the total data), the recall of plasma-derived exosome samples from mice with lung cancer and healthy mice was 98.1% and 97.0%, the positive predictive value (precision) was 96.4% and 98.5%, respectively (Figure 7E), AUC was 0.9879 (Figure 7F), and the overall accuracy was 97.5%. For the deep learning classification model constructed by CNN, the CVloss is 5.0%; for the same independent test set, the recall of plasma-derived exosome samples from lung cancer and healthy mice was 94.2% and 98.0%, the precision was 98.5% and 92.6%, respectively (Figure 7G), AUC was 0.9977 (Figure 7H), and the overall accuracy was 95.8%. The overall accuracy of these two models is very close to 98.7 % of the overall accuracy of individual cell-derived exosome samples, which indicates that animal plasma-derived exosomes can achieve the classification accuracy of cell-derived exosomes, and the microenvironment and various substances in the real blood samples of mice do not interfere with the proposed method. The above satisfactory animal experimental results fully verify the ability of the proposed method to predict EVs in real complex samples.

### Classification of the plasma-derived exosome samples from lung cancer patients and healthy people

#### SVM and CNN classification models

Finally, the ability of the proposed method to predict EVs was systematically evaluated using real clinical blood samples from lung cancer patients and healthy people (1500 and 1200 spectra respectively, a total of 2700 spectra). For the machine learning classification model constructed by SVM with optimized hyperparameters, the CVloss is 7.7%; for the independent test set (810 spectra, 30% of the total data), the recall of plasma-derived exosome samples from lung cancer patients and healthy people was 95.4% and 87.0%, the precision was 89.4% and 94.2%, respectively (Figure 7I), AUC was 0.9714 (Figure 7J), and the overall accuracy was 91.5%. For the deep learning classification model constructed by CNN with optimized hyperparameters, the CVloss is 8.3%; for the same independent test set, the recall of plasma-derived exosome samples from lung cancer patients and healthy people was 97.6% and 92.8%, the precision was 94.4% and 96.8%, respectively (Figure 7K), AUC was 0.9916 (Figure 7L), and the overall accuracy was 95.4%. Specifically, 439 of the 450 observations from the lung cancer patient class were correctly predicted by the CNN model to be in the lung cancer patient class (Recall = 97.6%), and 439 of the 465 observations predicted by the CNN to be in the lung cancer patient class were indeed from this class (Precision = 94.4%).

The classification results of these real clinical samples are very satisfactory, only slightly lower than the overall accuracy of individual cell-derived exosome samples, and similar to the classification results of animal models, indicating that the microenvironment and various substances in real human blood samples do not interfere with the proposed method. It is worth noting that these results are obtained on a relatively limited sample set. As the number of samples increases, the training set will be more representative and will cover more low-probability cases, so the generalization ability of the model will be better, and theoretically it will get better recall and precision. The satisfactory results of the above real clinical samples verify the clinical application potential of the proposed method.

#### Interpretation of the SVM machine learning model

**Figure 8:**
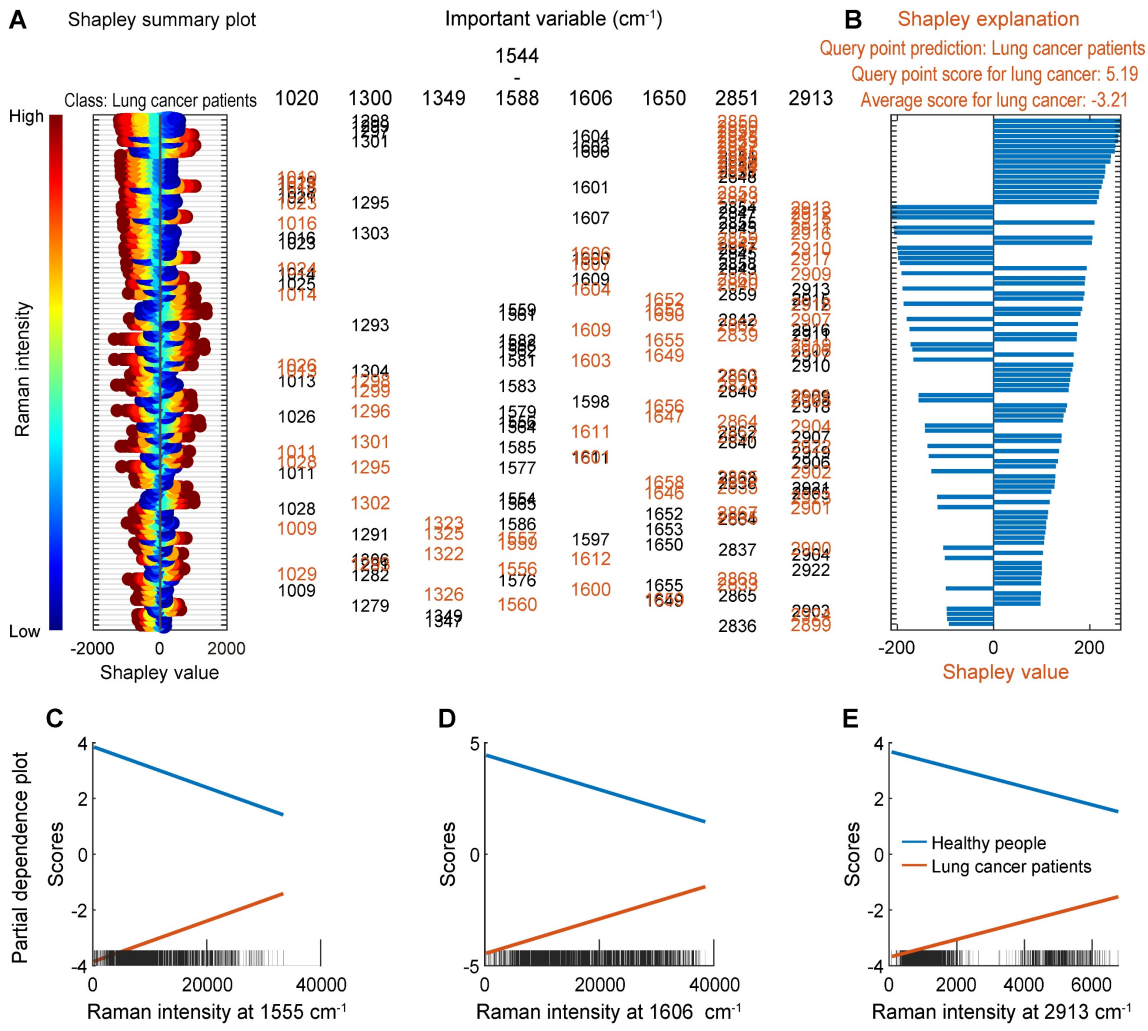
Interpretation of the machine learning model for the real clinical samples of lung cancer patients. (A) Shapley summary plot of the lung cancer class (Variables are marked in black). (B) Shapley explanation of a single query sample of the lung cancer class (Variables are marked in red). (C)-(E) PDPs for the three important variables.

In order to understand how the proposed machine learning classification model makes predictions, the Shapley values (SHAP) and partial dependence plot (PDP) were used to reveal how varialbes contribute to classification predictions, as shown in Figure 8. For local interpretation, the SHAP was calculated using the test set. Firstly, the distribution of the Shapley values of the lung cancer class was visualized using the swarm scatter chart, as shown in Figure 8A, the order of all variables was obtained based on the average of the absolute values of the Shapley values for all test samples. Then, the deviation of the predicted class scores from the average value was explained by the Shapley values of the variables of one single query samples, as shown in 8B. The SERS bands around 2851, 1300, 1606, 1020, 2913, 1650, 1555-1587, and 1349 cm^-1^ were identified as important variables for classification. These results are consistent with the results of PCA, and the order of importance of variables is further given. For global interpretation, PDPs were created for the important variables 1555, 1606, and 2913 cm^-1^, to explain how the proposed machine learning classification model makes predictions for the entire dataset, as shown in 8C, 8D, and 8E. These three subgraphs show the relationship between these important variables and the predicted class scores for the two classes, respectively. For example, the probability of lung cancer increases with the increase of the Raman signal at the variable 2913 cm^-1^.

## Conclusion

In this study, a precise classification method for lung cancer cell-derived EVs, as well as plasma-derived EVs from healthy and lung cancer patients, was established based on SERS combined with machine learning. By comparing the optical properties and basic properties (enhancement effect, uniformity, and repeatability), the suitable particle size of the SERS substrate AuNPs was determined to be 44.06 nm. The substrate and exosomes liquid were mixed evenly in the capillary and an unique liquid enhanced sampling technology was used to achieve a highly stable enhancement effect. Through the comparative study of the classification effects of classical machine learning algorithms (including DA, DT, SVM, and KNN), the CVloss of the SVM classification model (with hyperparameters optimized by Bayesian method) of A549 and BEAS-2B cell-derived exosomes was only 3.7%, and the overall accuracy of the independent test set reached 98.7%. The method was evaluated at multiple levels. The classification effects of SVM and CNN on five types of cell-derived exosomes were evaluated using HEK, HeLa, and HepG2 cell-derived exosomes as interferences, the CVloss was 0% and 0.4%, the overall accuracy of the test set was 99.5% and 100% respectively. The classification effects of SVM and CNN on four different proportions of mixed samples of A549 and BEAS-2B cell-derived exosomes were evaluated, the CVloss was 8.3% and 17.8%, the overall accuracy of the test set was 93.9% and 85.6% respectively. The lung cancer mice model was used to evaluate the application potential of the proposed method, the CVloss of the SVM and CNN model was 2.5% and 5.0%, and the overall accuracy of the test set of plasma-derived exosome samples from lung cancer and healthy mice was 97.5% and 95.8% respectively. Finally, the proposed method was used to discriminate plasma-derived exosome samples from lung cancer patients and healthy people, the CVloss of the SVM and CNN model was 7.7% and 8.3%, the overall accuracy of the independent test set was 91.5% and 95.4% respectively. The classification results of the above systems are satisfactory, which fully demonstrates the classification effect and application potential of the proposed strategy.

The machine learning-assisted liquid-enhanced SERS method can quickly and accurately classify lung cancer cell-derived or plasma-derived exosomes based on only a small amount of samples, with characteristics of small sample, high stability, and high accuracy. In practical applications, if there are less labeled data, it is more appropriate to use SVM for modeling; if there are a large amount of labeled data and high computing power, one can choose the CNN model. The proposed technology is expected to provide a rapid and precise strategy for early detection and monitoring of lung cancer. In addition, the results of PCA, SHAP, and PDP analysis also provided some biochemical information, in which the protein and metabolic levels of lung cancer cell were higher than those of normal cell, resulting in differences in the composition (lipids and proteins) of EVs.

## Supplementary Material

Supplementary methods, figures and tables.

## Figures and Tables

**Table 1:** Comparison of the classification performances of different ML algorithms.

Model	Overall accuracy (%)	CVloss (%)
LDA	96.9	4.9
QDA	96.9	3.7
Fine tree	96.3	4.3
Medium tree	96.3	3.1
Coarse tree	96.3	3.1
Linear SVM	98.8	0.6
Quadratic SVM	99.4	0.6
Cubic SVM	99.4	0.6
Fine gaussian SVM	67.9	30.9
Medium gaussian SVM	97.5	2.5
Coarse gaussian SVM	96.3	3.7
Fine KNN	98.1	1.2
Medium KNN	95.1	4.3
Coarse KNN	87.7	12.3
Cosine KNN	94.4	4.9
Cubic KNN	94.4	4.3
Weighted KNN	95.1	3.1
Boosted trees	53.7	46.3
Bagged trees	98.8	2.5
Subspace discriminant	92.0	6.8
Subspace KNN	98.1	1.8
RUS boosted trees	79.6	46.3

## Abbreviations

A549: Human non-small cell lung cancer cells
AUC: Area under curve
AuNPs: Gold nanoparticles
BEAS-2B: Lung epithelial cells
BT: Boosting tree
CNN: Convolutional neural network
CVloss: Classification error rate of the five-fold cross-validation of a model
DA: Discriminant analysis
DLS: Dynamic light scattering
DT: Decision tree
EF: Enhancement factor
ELISA: Enzyme-linked immunosorbent assay
EVs: Extracellular vesicles
HEK: Embryonic kidney cells
HeLa: Cervical cancer cells
HepG2: Liver cancer cells
KNN: K-nearest neighbor
LR: Logistic regression
LSTM: Long short-term memory
ML: Machine learning
MPLS: Morphologically weighted penalized partial least squares
NTA: Nanoparticle tracking analysis
PBS: Phosphate buffer saline
PBST: Phosphate buffered saline with tween 20
PCA: Principal component analysis
PDP: Partial dependence plot
R6G: Rhodamine 6G
ReLU: Rectified linear unit
RNN: Recurrent neural networks
ROC: Receiver operating characteristic
RS: Raman spectroscopy
SERS: Surface enhanced Raman spectroscopy
SHAP: Shapley values
SVM: Support vector machines
TEM: Transmission electron microscopy
WB: Western blot
WIO: Water immersion objective
