# Supplementary material for: Lung cancer diagnosis through extracellular vesicle analysis using label-free surface-enhanced Raman spectroscopy coupled with machine learning
Source: Theranostics. 2025 Jun 23;15(15):7545–66. doi: 10.7150/thno.110178 (PMC12315814; doi:10.7150/thno.110178)
Supplement: Supplementary file 1 — Supplementary methods, figures and tables. [file thnov15p7545s1.pdf]

Supplementary material for

Lung cancer diagnosis through extracellular vesicle analysis using label-free surface-enhanced Raman spectroscopy coupled with machine learning

Hai-Sha Liu[1], Kai-Wen Ye[2], Jun Liu[2], Jin-Kuang Jiang[1], Ying-Fang Jian[1], Dong-Mei Chen[1], Chao Kang[1*], Li Qiu[2*], Ya-Juan Liu[3*]

[1] School of Chemistry and Chemical Engineering, Guizhou University, Guiyang 550025, China

[2] Department of Thoracic Surgery and Oncology, the First Affiliated Hospital of Guangzhou Medical University, State Key Laboratory of Respiratory Disease & National Clinical Research Center for Respiratory Disease, Guangzhou 510120, China

[3] Guangzhou Municipal and Guangdong Provincial Key Laboratory of Molecular Target & Clinical Pharmacology, the NMPA and State Key Laboratory of Respiratory Disease, School of Pharmaceutical Sciences, Guangzhou Medical University, Guangzhou 511436, China

*Corresponding authors:

*E-mail: ckang@gzu.edu.cn (Chao Kang)

*E-mail: qiuli848@gzhmu.edu.cn (Li Qiu)

*E-mail: yjliu@gzhmu.edu.cn (Ya-Juan Liu)

Animal experiments of lung cancer and healthy mice

Animal test materials

C57 male mice were 6 to 8 weeks old, weighing 20 ± 2 g, and were housed in separate cages. The temperature was 20 - 24 °C, 12 h light-dark cycle, relative humidity was 40% - 70%, and the mice were adapted to feeding for 7 days before the start of the experiment. In natural light, eat and drink freely during the experiment.

Source of experimental animals: Beijing Weitong Lihua Laboratory Animal Technology Co., Ltd., SPF male C57 mice.

Mice modeling

After 7 days of adaptive feeding of mice, the mice were grabbed, the skin of the injection site was wiped with an alcohol cotton ball for disinfection, and 200 μL of Lewis lung cancer cell suspension was injected into the inoculation site (5×10^6 cells), and after the needle was withdrawn, the pinhole was gently pressed to confirm that there was no exudation, and then put it back into the cage box for normal feeding.

The mice tumors were measured with vernier calipers, and when the tumor diameter was greater than 10 mm, the two groups of mice were collected from the orbits, and each group of mice was recorded and photographed before sacrifice.

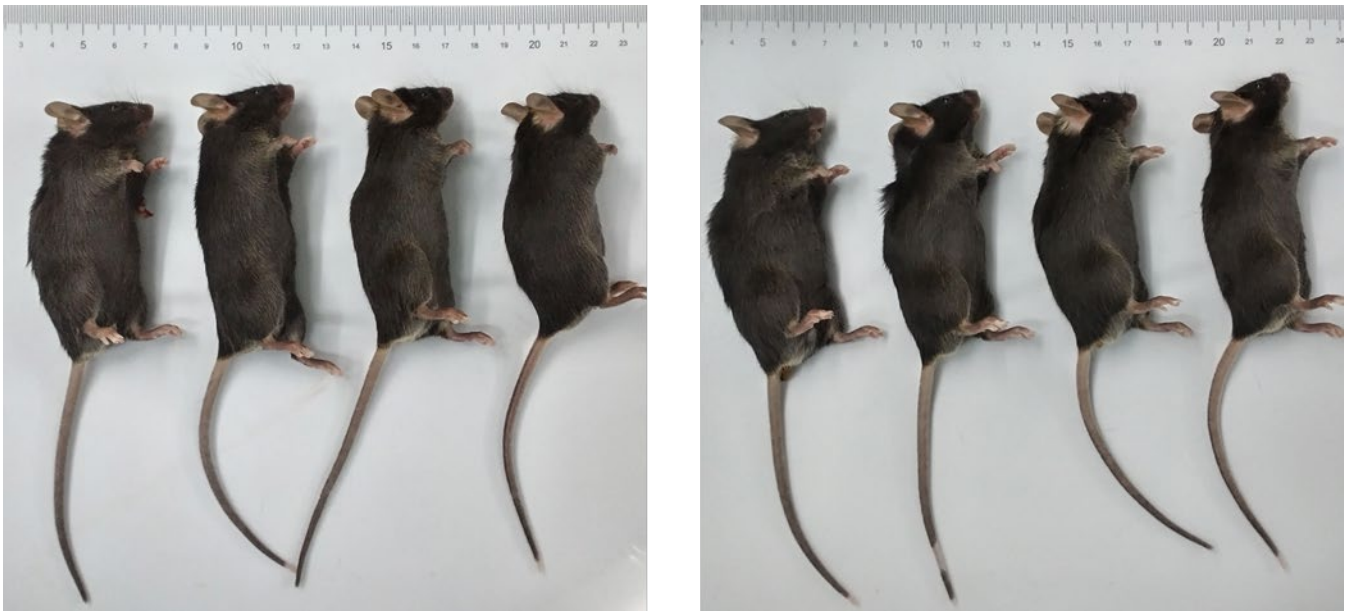

Figure S1 Healthy mice for controls.

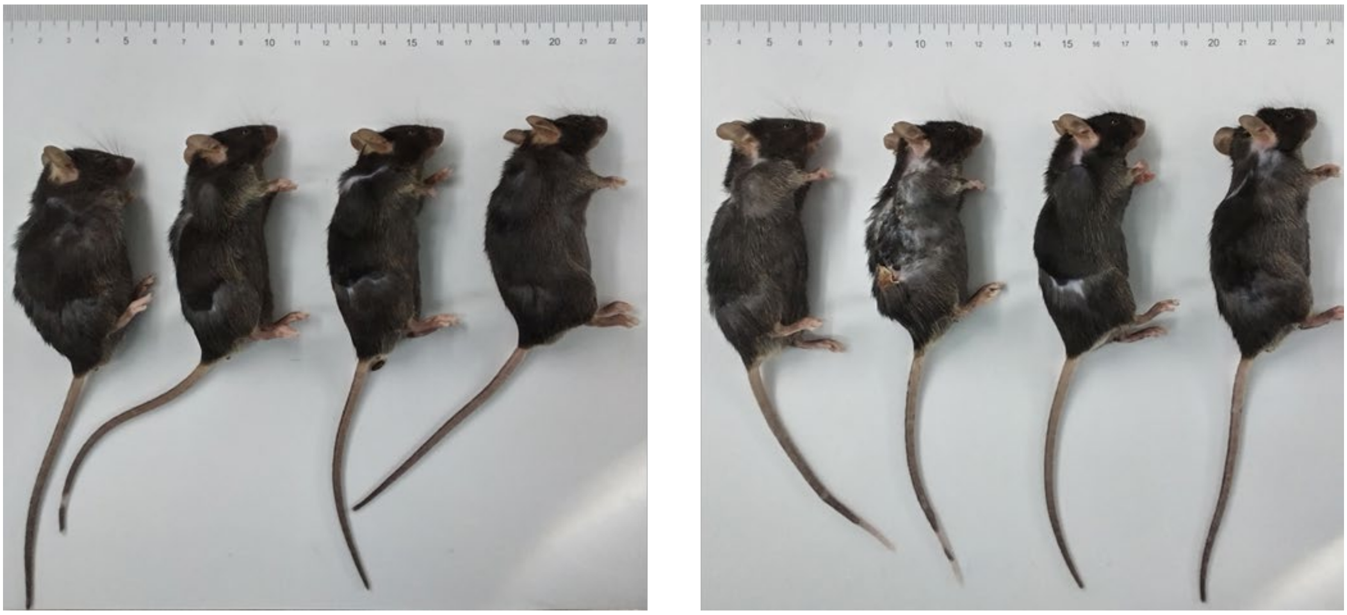

Figure S2 Mice injected with lung cancer cell suspension.

Table S1 Tumor size

**Constituencies**	**serial number**	**Short diameter of Tumor (mm)**	**Short diameter of Tumor (mm)**
Tumor	1	12.86	11.67
	2	14.76	10.87
	3	13.99	10.92
	4	8.73	8.72
	5	15.89	12.49
	6	9.86	9.11
	7	13.06	10.9
	8	16.61	11.03

Clinical sample of lung cancer patients and healthy people

Blood sample collection

Peripheral venous blood samples are drawn from patients and healthies. A 5 mL blood sample was collected using a vacuum blood collection tube (EDTA tube). Mix the anticoagulant tube by gently inverting it immediately after harvesting (5-8 times) to avoid vigorous shaking. Plasma samples were collected by centrifugation at 1500 rpm for 15 minutes.

Principal component analysis

PCA is one of the most widespread exploratory data analysis (unsupervised learning) algorithms [1, 2]. PCA decomposes the data matrix into score and loading matrices:

$$\mathbf{X}_{I\times J} = \mathbf{T}_{I\times A}\mathbf{P}_{J\times A}{}^{\mathrm{T}} + \mathbf{E}_{I\times J} = \mathbf{t}_1\mathbf{p}_1^{\mathrm{T}} + \mathbf{t}_2\mathbf{p}_2^{\mathrm{T}} + \cdots + \mathbf{t}_A\mathbf{p}_A^{\mathrm{T}} + \mathbf{E}_{I\times J} \qquad (1)$$

where I and J are the number of samples and variables, X is the data matrix composed of multivariate measurement data of I samples, E is the residual matrix, T is the score matrix of which the ath column vector ta represents the score of the ath principal component, and P is the loading matrix of which the ath column vector pa represents the loading of the ath principal component. PCA can reduce the dimensionality of a high-dimensional dataset with many variables to a low-dimensional dataset composed of only two or three principal components. On the basis of the score plot and loading plot of the first two or three principal components, clustering between samples in the original high-dimensional space can be visualized, and primitive variables contribute more to clustering [1].

Table S2 SERS peak assignment of EVs

Peak（cm^{-1}）	Assignment	Change	Ref
493	Glycogen	Increase	[3, 4]
645	N–H bending of amide V	New	[5]
741	O–CN bending of amide IV	Increase	[5]
1011	Breathing of benzene ring	Increase	[6]
1078	C–C and C^{ε}–N^{ζ} stretching of lysine	Increase	[7]
1163	C^{α}–C^{β} or C^{β}–C^{γ} stretching of valine	New	[7]
1221	Amide III (β-sheet)	Increase	[8]
1349	C^{α}–H bending and C^{α}–C stretching	Increase	[9]
1437	CH_2 bending of lipids	Increase	[10]
2913	C–H stretching of lipids and proteins	Increase	[11]

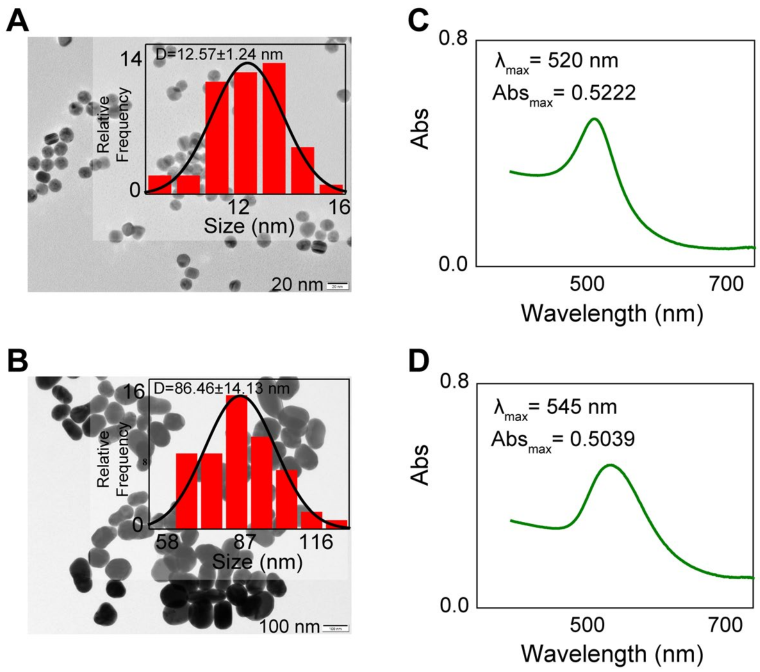

Fig S3 Characterization of gold nanoparticles of different sizes. (A) and (B) TEM of the AuNPs, inset shows particle size statistics. (C) and (D) show the visible spectra of the two gold nanoparticles.

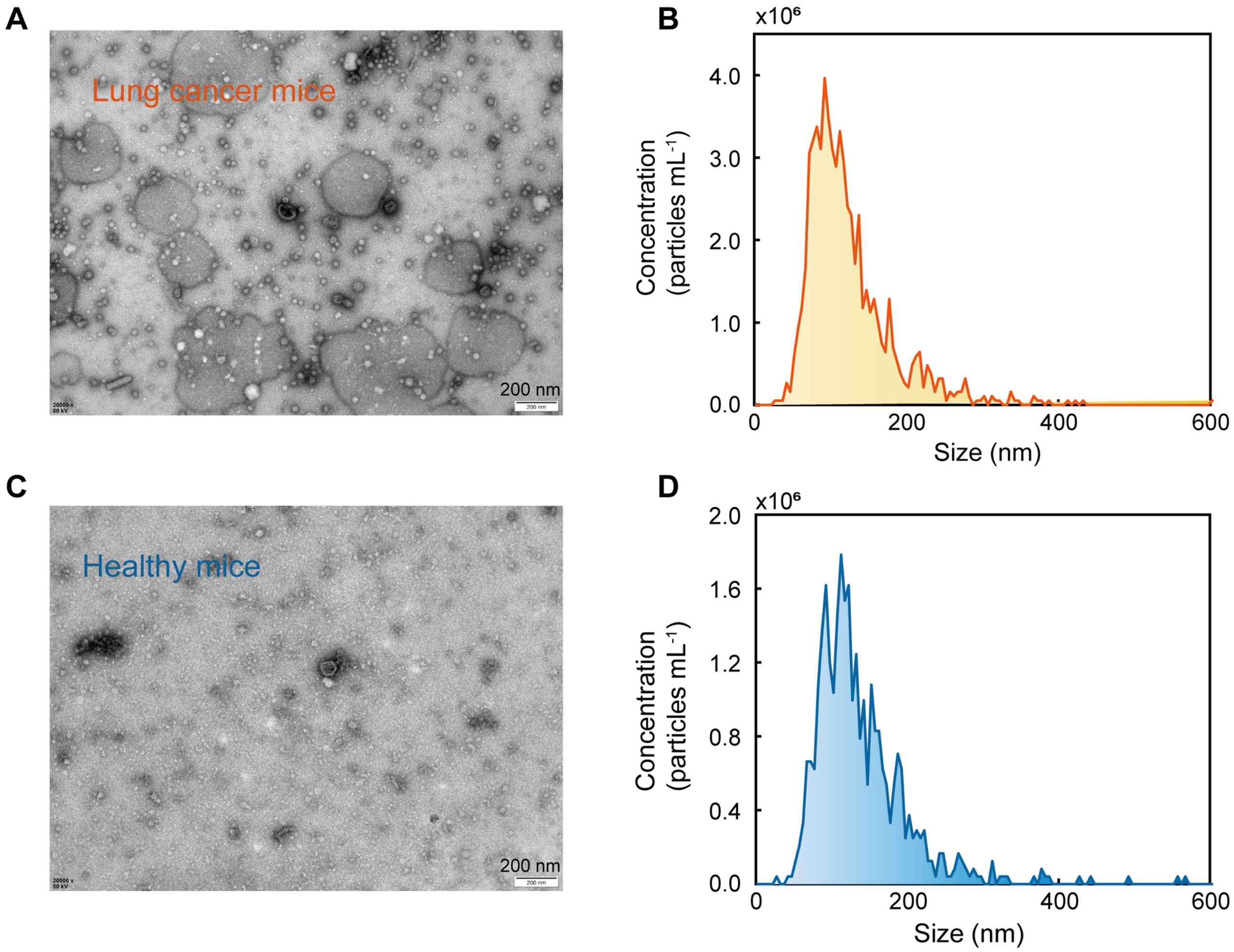

Figure S4 Characterization results of mice plasma-derived exosomes. (A) TEM results. (B) NTA results.

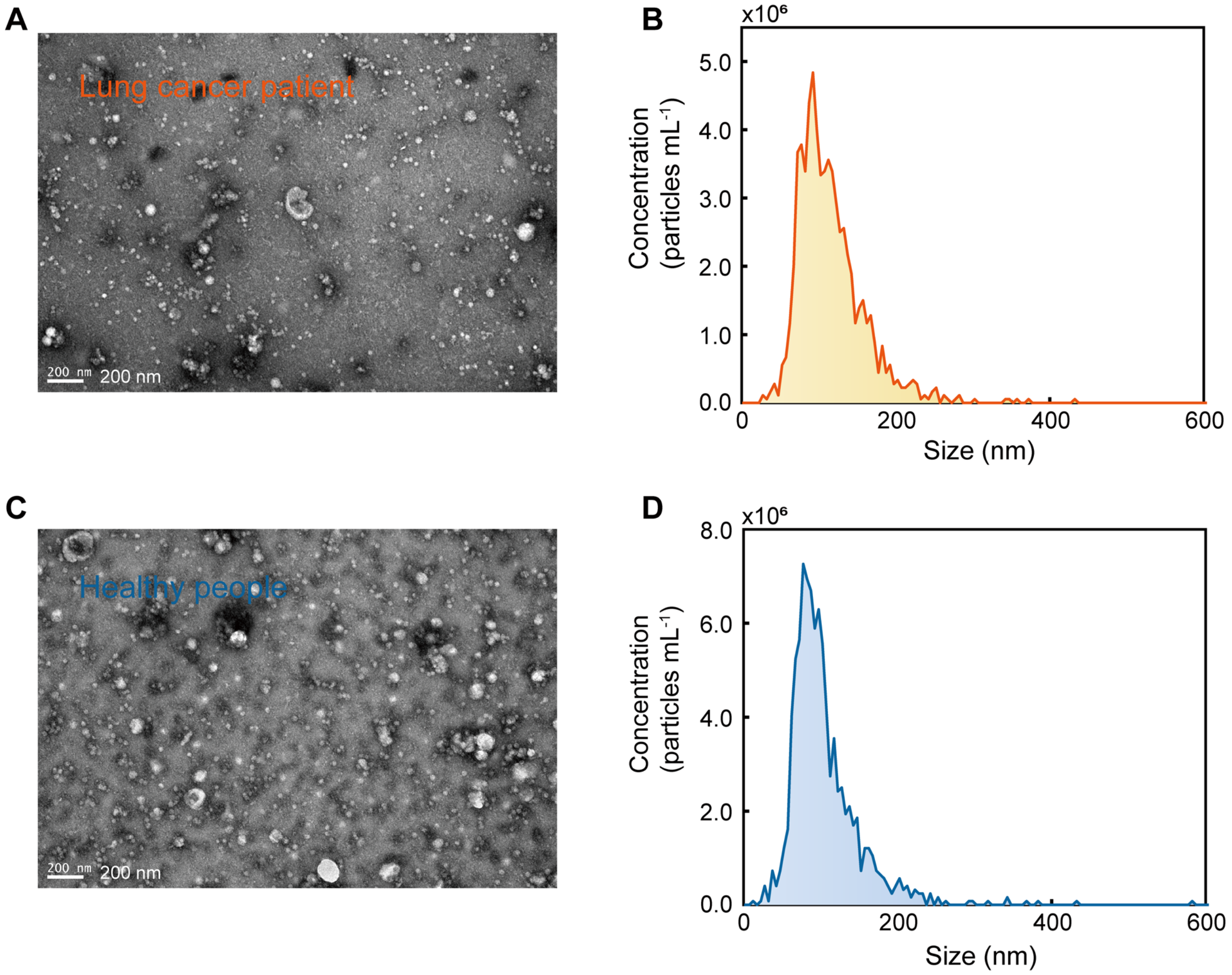

Figure S5 Characterization results of human plasma-derived exosomes. (A) TEM results. (B) NTA results.

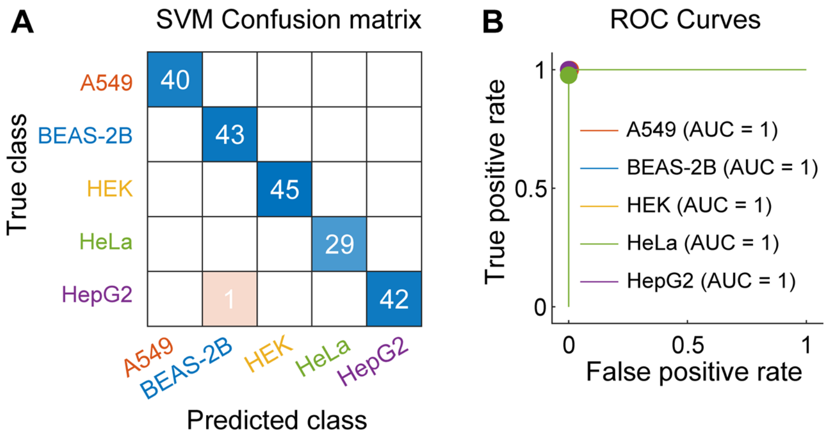

Fig S6 SVM model developed for predicting exosomes derived from five different cell lines. (A) Confusion matrix for independent test set. (B) ROC curve and AUC value for the independent test set.

References:

1. Brown SD, Tauler R, Walczak B. Comprehensive chemometrics: Chemical and biochemical data analysis. Amsterdam: Elsevier; 2009.
2. Ringnér M. What is principal component analysis? Nat Biotechnol. 2008; 26: 303-4.
3. Qian HY, Shao XG, Zhu YJ, Fan LC, Zhang H, Dong BJ, et al. Surface-enhanced Raman spectroscopy of preoperative serum samples predicts Gleason grade group upgrade in biopsy Gleason grade group 1 prostate cancer. Urol Oncol-semin Ori. 2020; 38: 601.e1-.e9.
4. Zhang H, Chen ZY, Wu JP, Chen N, Xu WJ, Li TH, et al. Laser stimulating ST36 with optical fiber induce blood component changes in mice: a Raman spectroscopy study. J Biophotonics. 2018; 11: e201700262.
5. Kuhar N, Sil S, Umapathy S. Potential of Raman spectroscopic techniques to study proteins. Spectrochim Acta A Mol Biomol Spectrosc. 2021; 258: 119712.
6. Unno M, Kikuchi S, Masuda S. Structural refinement of a key tryptophan residue in the BLUF photoreceptor AppA by ultraviolet resonance Raman spectroscopy. Biophys J. 2010; 98: 1949-56.
7. Overman SA, Thomas GJ. Raman markers of nonaromatic side chains in an α-Helix assembly: Ala, Asp, Glu, Gly, Ile, Leu, Lys, Ser, and Val residues of phage *fd* subunits. Biochemistry. 1999; 38: 4018-27.
8. Panikkanvalappil SR, James M, Hira SM, Mobley J, Jilling T, Ambalavanan N, et al. Hyperoxia induces intracellular acidification in neonatal mouse lung fibroblasts: real-time investigation using plasmonically enhanced Raman spectroscopy. J Am Chem Soc. 2016; 138: 3779-88.
9. Overman SA, Thomas GJ. Amide modes of the α-Helix: Raman spectroscopy of filamentous virus *fd* containing peptide ^{13}C and ^{2}H labels in coat protein subunits. Biochemistry. 1998; 37: 5654-65.
10. Qian HY, Shao XG, Zhang H, Wang Y, Liu SP, Pan JH, et al. Diagnosis of urogenital cancer combining deep learning algorithms and surface-enhanced Raman spectroscopy based on small extracellular vesicles. Spectrochim Acta A Mol Biomol Spectrosc. 2022; 281: 121603.
11. Qin YF, Lu XY, Shi Z, Huang QS, Wang X, Ren B, et al. Deep learning-enabled Raman spectroscopic identification of pathogen-derived extracellular vesicles and the biogenesis process. Anal Chem. 2022; 94: 12416-26.
